# The Moral Foundations of Vaccine Passports

**DOI:** 10.1007/s10551-023-05427-8

**Published:** 2023-05-21

**Authors:** Trisha Harjani, Hongwei He, Melody Manchi Chao

**Affiliations:** 1 Department of Psychology, University of Cambridge, Cambridge, CB2 3EB UK; 2 Alliance Manchester Business School, The University of Manchester, Manchester, M13 9SS UK; 3 Department of Management, School of Business and Management, The Hong Kong University of Science and Technology, Clear Water Bay, Kowloon, Hong Kong S.A.R.

**Keywords:** Vaccine passport, COVID-19 pandemic, Non-pharmaceutical intervention, Business policy, Moral foundations

## Abstract

The debate around vaccine passports has been polarising and controversial. Although the measure allows businesses to resume in-person operations and enables transitioning out of lockdown due to the COVID-19 pandemic, some have expressed concerns about liberty violations and discrimination. Understanding the splintered viewpoints can aid businesses in communicating such measures to employees and consumers. We conceptualise the business implementation of vaccine passports as a moral decision rooted in individual values that influence reasoning and emotional reaction. We surveyed support for vaccine passports on a nationally representative sample in the United Kingdom in 2021: April (*n* = 349), May (*n* = 328), and July (*n* = 311). Drawing on the Moral Foundations Theory—binding (loyalty, authority, and sanctity), individualising (fairness and harm), and liberty values—we find that individualising values are a positive predictor and liberty values a negative predictor of support for passports, suggesting adoption hinges on addressing liberty concerns. Longitudinal analysis examining the trajectory of change in support over time finds that individualising foundations positively predict changes in utilitarian and deontological reasoning over time. In contrast, a fall in anger over time predicts increased support towards vaccine passports. Our study can inform business and policy communication strategies of existing vaccine passports, general vaccine mandates, and similar measures in future pandemics.

## Introduction

The proposal and implementation of vaccine passports stirred heated debates around the world amid the COVID-19 pandemic ( Martuscelli & Roberts, 2021; Merritt, 2021; Associated Press, 2021). Among a host of responses to the recent pandemic (e.g., social distancing, handwashing) that have been moralised (Pavlović et al., 2022; Prosser et al., 2020), the implementation of vaccine passports has drawn much attention. This pandemic countermeasure involves using a—typically digital—passport displaying proof of vaccination, sometimes substituted by a negative test or evidence of infection, to enter a domestic venue (such as a workplace, restaurant, or concert) or another country. The measure is significant beyond COVID-19 and requires attention because it presents an ethical challenge within the workplace, given its potential to influence business activity and trigger significant concerns about limiting freedoms. We propose that the optimal policy design involves understanding the factors predicting support for the vaccine passport, as it would enable businesses to deploy tailored messaging. As such, we focus on individuals’ receptiveness to vaccine passports and investigate determinants of support.

The backlash to vaccine passports was widespread and polarising, prompting petitions and parliamentary debate in the UK (UK Government & Parliament, 2021), emergency legal action in the U.S. (The Economist, 2021), protests in Canada (Lindeman, 2022), and across Europe (Associated Press, 2022). However, vaccine passports were welcomed by others (Holmes & Kierszenbaum, 2021), and polling showed that support for the vaccine passport is highly variable around the world (Kirk, 2022).

Drawing from media and political debates (BBC Grossin et al., 2021; Hare, 2021; News, 2021a), we identified three overarching points of concern with the use of vaccine passports: that they could (1) do harm by excluding and discriminating against the unvaccinated, (2) violate basic freedoms and, (3) violate data privacy. These considerations suggested that one’s level of support towards vaccine passports can be understood as a moral decision rooted in individual moral values. This interpretation is based on an observed intuitive mapping between the objections to a vaccine passport and the three groupings of moral values, as proposed by Moral Foundations Theory, formulated as the individualising foundations (harm, fairness), binding foundations (purity, authority, and loyalty) and the foundation of liberty.

Moral Foundations Theory (MFT) is a pluralistic, intuitionist model of moral and psychological processes intending to capture the entirety of core moral values (Graham et al., 2011). Individual ratings on each foundation represent the extent to which one draws upon the value in moral decision-making. As Graham et al. (2011) outline, the harm/care foundation is concerned with the avoidance of suffering, the fairness/cheating foundation with issues of justice and equitable distribution, the loyalty/betrayal foundation with a preference for one’s in-group, the purity/degradation foundation with a preference for maintaining the sanctity of ‘natural’ bodily and environmental states, and the authority/subversion foundation with a preference for maintaining traditional social hierarchies and deferring to those in power. A sixth additional foundation of liberty that prizes individual freedoms and rights above all other foundations has also been proposed (Iyer et al., 2012).

Estimating the moral bases of support for a vaccine passport has significant ramifications for policy setting at the workplace and national level. As prior studies demonstrate, it can be effective to communicate moral issues with rhetoric congruent with one’s endorsement of moral foundations. Feinberg and Willer (2013) found that reframing environmental appeals using purity-based argumentation (climate change has violated the sanctity of the environment) increased pro-environmental attitudes for individuals endorsing the purity foundation (Dickinson et al., 2016). The strategy’s efficacy has been replicated for climate appeals (Kidwell et al., 2013), political persuasion (Day et al., 2014; Feinberg & Willer, 2015), and mask-wearing (Kaplan et al., 2021).

Extant research focusing on the vaccine passports has qualitatively investigated attitudes (Stead et al., 2022), tested interventions to boost support (Sotis et al., 2021) and examined the relationship between vaccine inclination and vaccine passports (de Figueiredo et al., 2021). The current paper would contribute to the nascent work of understanding public attitudes towards vaccination measures in general and toward business adoption of such measures in particular. To our knowledge, this is the first study to conceptualise and investigate the decision as a moral one. Uses of vaccine passports beyond COVID-19 exist, including yellow fever vaccine mandates for international travel or the requirement in the U.S. that schools impose mandatory vaccinations for kids (Skinner, 2017). Although prior studies have researched attitudes toward vaccination using moral foundations (Amin et al., 2017; Hornsey et al., 2018; Reimer et al., 2022) and other moral indicators (Betsch & Böhm, 2018; Betsch et al., 2015; Rossen et al., 2019), we contend that an understanding of vaccine passports necessitates an independent investigation especially as preliminary research demonstrates that since the COVID-19 pandemic, individuals in the UK have a higher perceived risk towards vaccines in general (Gallant et al., 2021; Yu et al., 2020). Crucially, previous literature that examined attitudes toward vaccines conceptualised vaccination as a personal and voluntary decision, whilst we investigate attitudes in a situation where vaccinations might grant increased mobility and access to society.

## Business Implications of COVID-19 Passport

Overall, businesses would need to account for at least two potential ramifications of implementing a vaccine passport: reputational damage and changes in employee sentiment. Understanding moral foundations can reduce the risk of companies, public organisations, or governments fracturing their relationship with employees, customers, and the general public that hold polarised attitudes. The implementation of a vaccine passport, or a similar condition of entry into the workplace, could damage branding through the exclusion of a class of employees, affecting a company’s reputation (Weber Shandwick, 2020). As Kong and Belkin (2021) show, employees can feel neglected if they experience a violation of the ‘psychological contract’ with their employer. Excluded employees could also feel discriminated against, potentially threatening a sense of belonging (Gibson, 2020), and risking lowered wellbeing and productivity (Greenhaus et al., 1990; Mor Barak et al., 1998; Schaufeli et al., 1996). Previous studies also note how moral foundations are likely to affect followers’ perceptions of leader behaviour. Specifically, a higher sensitivity for moral harm would imply that behaviours based on the harm foundation will be perceived as ethical and violations as unethical (Weaver et al., 2014). A more nuanced understanding of the moral support for vaccine passports can thus be insightful for communication practitioners.

Vaccine passports have been implemented both domestically and internationally. We anticipate that the business implications for both uses of the vaccine passport would be similar, varying only in strength, where the effect of moral values would have a stronger effect on support for domestic passports. Given vaccine mandates are a relatively established norm (e.g., yellow fever) as a requirement to enter many countries (Public Health Scotland, 2022), there is likely to be more baseline acceptance for international vaccine passports. Media reports and polling is suggestive of this, as opposition to vaccine passports is predominantly focused on domestic usage (Associated Kirk, 2022; Associated Press, 2022). As such, although international vaccine passports have also faced some backlash (Voigt et al., 2021), domestic passports are likely to be more susceptible to scrutiny and would likely require more caution in their communication and implementation.

## Compliance and Support for COVID-19 Measures

The body of research directly examining attitudes to vaccine passports is limited. Therefore, we briefly summarise research investigating compliance with and support for other measures to curb the spread of COVID-19. Non-moral predictors of social distancing behaviour include efficacy, age, and perceived behavioural control (Clark et al., 2020; Das et al., 2021; Ozdemir et al., 2020; Yu et al., 2020). Mask-wearing has been associated with higher perceived marketplace influence (how much you think others will wear a mask if you wear one), being older, less educated, and more concerned about the possibility of infection (Asri et al., 2021; Barceló & Sheen, 2020; Schneider & Leonard, 2021). Fear of COVID-19 was the strongest predictor of social distancing and hand hygiene in one study (Harper et al., 2021). Other predictors, including political affiliation (Clinton et al., 2021; Deane et al., 2021), trust in science (Plohl & Musil, 2020), confidence in healthcare systems (Chan et al., 2020), and economic factors (Wright et al., 2020), also have substantial impacts on compliance to COVID-19 related measures.

Moral predictors of compliance with COVID-19 guidelines included moral foundations and moral beliefs. Chan (2021) finds that valuing care and fairness foundations (individualising) increases the odds of complying with all COVID-19 measures (staying at home, mask-wearing, and social distancing) while valuing moral purity decreases the odds of mask-wearing and social distancing. This is consistent with research that finds endorsing moral fairness is associated with adherence to health guidelines (Syropoulos & Markowitz, 2021) and that higher valuation of the individualising foundations (harm and fairness) is associated with perceiving violations of health guidelines as less morally permissible (Bruchmann & LaPierre, 2021). Another study finds that a sense of moral obligation to comply with pandemic countermeasures is also associated with compliance (Kuiper et al., 2020; van Rooji et al., 2020).

## Theoretical Framework and Hypotheses Development

We use MFT to operationalise the individual moral bases of support for vaccine passports as it is one of the leading psychological theories used to account for differences in moral attitudes towards topical societal issues (Koleva et al., 2012). Indeed, Graham et al. (2013) note its usefulness in understanding divergent attitudes towards same-sex marriage, abortion, torture, pro-environmental attitudes (Dickinson et al., 2016; Feinberg & Willer, 2013); immigration (Baldner & Pierro, 2019; Chung et al., 2016; Grover et al., 2019), pro-social behaviour (Jancenelle et al., 2018; Nilsson et al., 2020; O’Grady & Vandegrift, 2019; Süssenbach et al., 2019) and compliance with COVID-19 measures (Bokemper et al., 2022; Chan, 2021; Bruchmann & LaPierre, 2021). MFT is, therefore, highly applicable to issues that have both moral and political considerations, such as the COVID-19 vaccination (Albrecht, 2022; Sharfstein et al., 2021) or vaccine passports (The Economist, 2021). Furthermore, due to the centrality of the liberty foundation to ethical debates (Thornton et al., 2022) about vaccine passports (Grossin et al., 2021), we also examined the impact of the liberty foundation in this study.

One of MFT’s classic findings is that liberals and conservatives vary in the extent to which they rely on particular sets of moral foundations, and this can help explain the divergence in their reactions: liberals tend to rely on the individualising foundations (harm and fairness) whereas conservatives tend to draw upon the binding foundations (loyalty, purity, and authority) (Graham et al., 2009; also see Kivikangas et al., 2021 for meta-analytic results). Iyer et al. (2012) also put forward a third moral profile of libertarians, distinct from liberals and conservatives in their supreme regard for freedom.

As alluded to previously, we observed approximate mapping between COVID-19 vaccine passport discourses and MFT’s moral profiles. Firstly, we expect that concerns of harm towards minorities (care foundation) and unfair discrimination (fairness foundation) represent a violation of individualising values. Critics claim a vaccine passport would create a two-tier society by granting vaccinated citizens greater access to parts of social and economic life (Cave et al., 2021; BBC, 2021a), with some questioning whether it violates basic human rights (Hall & Studdert, 2021). Opponents of implementing vaccine passports have expressed concern that it would exacerbate existing inequalities given the disproportionate impact of COVID-19 on minorities (Kalla, 2021) and a slower uptake in vaccination among certain ethnic groups (Kennedy, 2021; Sesa et al., 2021). Secondly, those with liberty-based concerns claim citizens are coerced into receiving a vaccine by providing vaccinated citizens with greater access and benefits (Allegretti, 2021; Cohen, 2021; Osama et al., 2021). Others have also raised questions about data management and privacy (Lee et al., 2021) as the disclosure of health data can breach the European Convention of Human Rights (Groppo, 2021). We expect an individual’s liberty foundation can capture these concerns. Despite formal expressions of liberty as a central concern to pandemic countermeasures (Gostin & Hodge, 2020), previous studies have not examined the liberty foundation as an antecedent to COVID-19 measures. Liberty concerns also united protestors across the political spectrum in their opposition to vaccine passports (Grossin et al., 2021). We thus expected the following:

### Hypothesis 1

Individualising foundations (harm, fairness) negatively predicts support for domestic and international vaccine passports.

### Hypothesis 2

The liberty foundation negatively predicts support for domestic and international vaccine passports.

Prior research has repeatedly found a negative association between the purity foundation and attitudes to vaccination in general (Amin et al., 2017; Rossen et al., 2019), as well as actual COVID-19 vaccination rates (Reimer et al., 2022). Thus, a less favourable perception of vaccines (due to purity) may imply lower support for passports. However, for two key reasons, we do not make specific predictions regarding the direct or indirect effect of the binding foundations on support for vaccine passports. Firstly, our hypotheses were formed by synthesising discourses in the media with prior relevant literature. As MFT seeks to identify the core values that shape moral reasoning and judgement, one may expect a relationship between foundations and attitudes (such as support for vaccine passports) in which the relevant issue violates that foundation or foundations. Although vaccination itself appears to violate the purity foundation, there is no clear evidence that attitudes toward vaccination can be extrapolated to the domain of vaccine passports. Given that the purity foundation appears to influence vaccination attitude through eliciting disgust, this relationship is more implicit because vaccination appears to elicit disgust in a visceral sense via the perception that vaccines are “unnatural” (Amin et al., 2017; Rossen et al., 2019). It is unclear if such an effect can be extrapolated to vaccine passports, a contentious issue due to its potential social and economic ramifications. Second, existing findings about binding foundations suggest that the prediction of binding foundations might not be straightforward as Rossen et al. (2019) noted that both higher endorsement of the purity foundation and lower valuation of the authority foundation predicted vaccination rejection.

Although the idea of “proof of vaccination” has been around, the implementation of vaccine passports framed as an entry requirement for domestic venues and international travel is new. Thus, we did not expect one-to-one relationships between moral foundations and support towards vaccine passports. As MFT and its precursor, the Social Intuitionist Model, suggest, foundations are the primary guiding intuitions that determine moral evaluations, with cognitive and rational arguments subsequently employed to justify the evaluations (Graham et al., 2013; Haidt, 2001). We propose that there are two predominant cognitive pathways individuals employ when making moral foundation-based judgements: a deontological (rule-based) and a utilitarian (consequence-based) path. This conceptualization is consistent with media debates: proponents of passports argue that the vaccine is low-risk and low-cost, outweighed by significant societal benefits (Sahakian et al., 2021). This utilitarian argument is supported by modelling showing that with mandatory vaccine passports in the U.K., cases and deaths could have been reduced by 30% (Sleat et al., 2021). Deontological concerns about health data privacy (Holland et al., 2021) and intrinsic violations of freedom (Martuscelli & Roberts, 2021) were also widely expressed in the vaccine passport discourse.

The proposed pathways are also consistent with the theorising of moral reasoning as rule-based and/or consequence-based (Conway & Gawronski, 2013). Kohlberg also differentiated the post-conventional level of moral reasoning in this way, dividing it into a “social contract orientation” grounded in utilitarian reasoning and a “universal-ethical-principle orientation” aligned with deontological styles (Kohlberg, 1975). Some have drawn parallels between the post-conventional level and moral foundations and found a negative association with binding foundations (Glover et al., 2014) and a positive one with individualising foundations (Baril & Wright, 2012). However, these studies do not discriminate between the orientations outlined by Kohlberg. Moreover, a recent study found that those high on individualising foundations were also high idealists, “endorsing both reliance on moral standards and striving to minimize the harm done to others” (O’Boyle & Forsyth, 2021). Meanwhile, Iyer et al. (2012) observed a negative association between liberty and idealism, finding that libertarians are more relativist (do not necessarily endorse a utilitarian or deontological stance). Thus, although the media, extant empirical work and theory suggest the distinctiveness of the reasoning styles, they do not appear to be mutually exclusive and associated with a particular moral profile. We, therefore, explore the relationships as an open research question rather than propose exclusive associations.

We further argued that emotional reactions to the passport would play an important role in the relationship between moral foundations and vaccine passports. Graham et al., (2013) note that emotions manifest as reactions to the violations of moral foundations. Specifically, violation of the purity foundation elicits disgust (Horberg et al., 2009; Rozin et al., 1999, 2008), whereas violations of harm and fairness values show mixed effects on anger and contempt (Gutierrez & Giner-Sorolla, 2007; Steiger & Reyna, 2017). Others found that anger and disgust interact to influence moral judgements (Salerno & Peter-Hagene, 2013). Importantly, the violation of moral foundations in general often triggers a mix of negative emotions, collectively termed “moral outrage” (Brady & Crockett, 2019; Brady et al., 2020). Consistent with this idea, recent research investigating the somatosensory response finds an association between a general “moral upset” and foundation violations (Atari et al., 2020). We predict that the negative effects of individualising and liberty foundations on support for vaccine passports are mediated by moral emotion (Hypotheses 3 and 4). We focus specifically on anger as the moral emotion in this study, given that this is the most prevalent emotional reaction reflected in media discourses (BBC, 2021b; Davidson, 2021; Muldoon, 2021; Sommerville, 2021). Comparatively, we largely expected moral disgust to play a distal role through vaccine attitudes though this is not formally hypothesised or tested.

### Hypothesis 3

The effect of individualising foundations on support for domestic and international vaccine passports is mediated by anger reactions, whereby increased anger predicts lowered support.

### Hypothesis 4

The effect of liberty foundations on support for domestic and international vaccine passports is mediated by anger reactions, whereby increased anger predicts lowered support.

We conducted a study in the United Kingdom exploring the relationship between moral foundations and support for both international and domestic passports in April 2021, as the U.K. government proposed the vaccine passports in early 2021 (Cabinet Office, 2021, p. 40). We tested the hypothesised relationships between moral foundations and vaccine passport support (Hypotheses 1 and 2), as well as the mediating role of anger (Hypotheses 3 and 4). We also explore the mediating role of moral reasoning as an open question.

## Methods

To gauge the relationship between individual moral foundations and attitudes towards vaccine passports, we ran a three-wave longitudinal study in the United Kingdom. Each wave was spaced approximately 1 month apart from April to July 2021. At the time of the study, vaccine passports received heavy media coverage during April and May 2021 as they were being proposed and considered by the U.K. government (UK Government & Parliament, 2021), which coincided with the vaccine roll-out. As such, we anticipated that attitudes might shift over time due to both exogenous and endogenous factors. Therefore, we conducted this longitudinal study with moral foundations measured once as individual differences in the first survey. The mediators and dependent variables were measured three times on the same participants over time to observe the evolution of attitudes. Longitudinal analysis conducted on the change in attitudes was exploratory as no study, to our knowledge, has assessed the relationship between moral foundations and attitude change over time. This also allowed for repeated testing of our four cross-sectional hypotheses above. For simplicity, Hypotheses 1 to 4 and the exploratory mediating effect of moral reasoning are tested at each wave and reported together in the results section.

### Data and Sample

Our longitudinal survey was administered using Prolific Academic (Palan & Schitter, 2018). We ran our first survey on 30 April 2021, recruiting a nationally representative sample of 360 adults from the United Kingdom.^1^ Data from 11 participants were excluded based on the following criteria: incomplete surveys (*n* = 7), no consent (*n* = 2), invalid responses^2^ (*n* = 1) and failing the attention check (*n* = 1), leaving a final sample of 349. Participants were paid 1.05 GBP. The first follow-up was sent to all 349 participants on 28 May 2021, garnering 330 responses, of which incomplete surveys were excluded (*n* = 2). The second follow-up was administered to all 328 who completed the first two surveys on 2 July 2021, and we received 311 complete responses. Participants were paid an additional 0.50 GBP and 0.82 GBP for the second and third surveys. The final sample of 311 observations (89% retention) consisted of 48% female (52% male) with an average age of 48. The full demographic breakdown is available in Appendix 1.

### Measures

#### Moral Foundations

In the first survey, we measured participants’ moral foundations using the 30-item moral foundations questionnaire (Graham et al., 2011), measuring how much participants endorsed each of the five foundations. The endorsement was measured using two types of questions. Participants first rated the relevance of 15 statements of moral judgements on a scale of 1 to 6 (1 = “not at all relevant”, 6 = “extremely relevant”). For example, a statement measuring fairness endorsement required participants to specify how relevant “whether or not someone acted unfairly” is to their moral decision-making. Participants then expressed their level of agreement with 15 statements on a scale of 1 to 6 (1 = “strongly disagree”, 6 = “strongly agree”). For example, participants indicated their agreement with the statement, “I think it’s morally wrong that rich children inherit a lot of money while poor children inherit nothing”. Graham et al. (2011) note that each of the foundations falls under the umbrella of ‘individualising’ (care, fairness) (*α* = 0.83) or ‘binding’ (purity, authority, loyalty) (*α* = 0.88) foundations. The moral foundations measure also included liberty, a newer foundation studied alongside the five original foundations (Iyer et al., 2012). This scale was nine items long, with two items measured on the relevance scale and seven items on the abovementioned agreement scale. The moral foundations measure was treated as a trait variable and only measured in the first survey.

#### Support for Vaccine Passports

Our primary dependent variable was in support for and attitude towards vaccine passports. Participants were first asked to read a generic description of the vaccine passports being proposed, which included key details, “Vaccine passports …granted by the government to every citizen that receives a vaccine…allows the holder to move freely within a city, state, country or even cross-countries” (full description in Appendix 2). The survey then required participants to, in a random order, rate statements about domestic and international passports.

No psychometrically valid scale measuring attitudes and support for vaccine passports was developed at the time of study design. Thus, we drew on some of the extant literature (Hall & Studdert, 2021; Lewandowsky et al., 2021) and arguments in the media (BBC Cave et al., 2021; Grossin et al., 2021; News, 2021a). This resulted in a 39-item scale measuring support, attitudes, perceived ethicality, cognitive judgments about the passports and emotional reactions towards the passports. The same scale was used for international and domestic passports. It was thus presented to participants two times, once with a description for international use (to cross borders) and once with a description for domestic use (for use in hotels, concerts, sports stadiums, theatres, and nightclubs). All Cronbach alpha values per wave are reported in Appendix 2 (Taber, 2018).

Global support was measured with three items, for example, “If a vaccine passport were to be implemented for domestic (international) use, I would support a government proposal for the passport”. Ratings were on a scale of 1 to 6 (1 = Strongly Disagree to 6 = Strongly Agree).

#### Cognitive and Emotional Appraisals

Statements concerning the judgement of vaccine passports measured two cognitive pathways: deontological (rule-based) and utilitarian (consequence-based). Participants indicated agreement with five statements measuring deontological reasoning, such as “If a vaccine passport were implemented for domestic (international) use, I believe it would be a discriminatory measure”. Similarly, participants rated their agreement with seven statements measuring utilitarian reasoning, such as, “If a vaccine passport were implemented for domestic (international) use, I believe it would help drive business recovery”. The scale also measured state emotional reactions to introducing a vaccine passport. Based on a prior adaptation by Fredrickson et al. (2003) of the Differential Emotions Scale (Boyle, 1984) measuring an individual’s emotional reactions to 9/11, we measure anger in response to the vaccine passports. Specifically, participants were asked to rate, on a scale of 1 to 5 (1 = Not at all, 2 = Not very much, 3 = A little, 4 = Somewhat, 5 = A lot, 6 = Very Much), their emotional reaction if the vaccine passports were implemented. The dependent variables were measured at all three timepoints.

### Confirmatory Factor Analysis

As we developed a new scale, we ran exploratory factor analyses to view the factor loadings for each item within the 12 cognitive judgement items. A priori, the scale was designed with seven items loading onto a ‘deontological’ factor and five loading onto a ‘utilitarian’ factor. Therefore, we ran a principal component analysis with varimax rotation, revealing a 2-factor solution for domestic and international vaccine passports. We excluded two items as they displayed cross-loadings above 60%. Table 1 shows the final factor loadings from Wave 1, representing utilitarian and deontological reasoning with 6 and 4 items, respectively. These variables are employed in subsequent analyses below.

**Table 1:** Confirmatory factor loadings

	Domestic		International	
	Utilitarian reasoning	Deontological reasoning	Utilitarian reasoning	Deontological reasoning
Item 22	0.86		0.83	0.43
Item 24	0.80		0.82	
Item 26		0.82		0.83
Item 27		0.92		0.91
Item 28		0.84		0.84
Item 29		0.90		0.90
Item 30	0.85		0.87	
Item 31	0.85		0.88	
Item 32	0.90		0.91	
Item 33	0.90		0.88	

See Appendix 2 for Items

### Control Variables

Participants were asked to rate how positively they view vaccines, as well as how safe, effective, reliable, and important they judge vaccines to be. The measure was adapted from Freeman et al. (2020) and formed an overall measure of vaccine attitudes at each wave. All surveys also tracked the vaccination status of participants (Department of Health and Social Care & Hancock, 2021).

The first survey also gathered demographic information, including the level of trust in vaccines, doctors and science, perceived risk of COVID-19, how worried they are about suffering from ‘long covid’,^3^ whether they lived with a medically vulnerable person, are a carer, and have recovered from covid. Other variables recorded include age, gender, political leaning (left-wing, centre, or right-wing), support for a UK political party (if any), education level, ethnicity, religion, the importance of religion, household income, household size and occupation. Where possible, measures were from UK census data (Office for National Statistics, 2020).

## Analysis and Findings

The analysis presented below is cross-sectional (at each wave) and longitudinal, as the study was conducted over three time-points.

### Cross-sectional Analysis

**Fig. 1:**
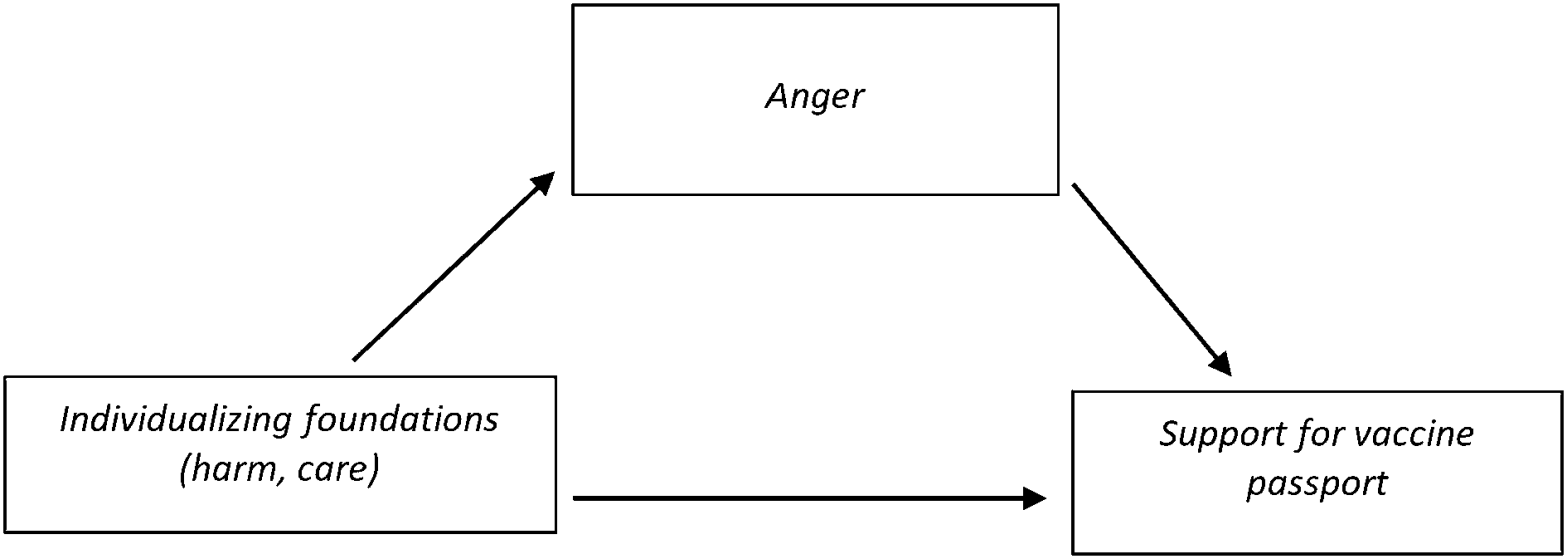
Path model illustrating the model testing Hypothesis 1 and 3 using SPSS PROCESS model 4

To test the hypotheses and our exploration of moral reasoning as a mediator simultaneously, for each wave of the study, we employed PROCESS model 4 (Hayes, 2014; see Fig. 1 above for conceptual model). This allowed us to test the mediated relationships in Hypotheses 3 and 4. Though we did not hypothesise the specific directional effect of cognitive reasoning (utilitarian or deontological), we included them as mediators.

#### Domestic Vaccine Passports

Using path modelling for the first (*n* = 349), second (*n* = 328), and third (*n* = 311) waves, we find no direct effects of either moral foundation (individualising, binding, or liberty) on support for domestic passports. However, we find significant total and indirect effects of individualising and liberty foundations, supporting mediated relationships (Hayes, 2014).

The positive effect of individualising foundations on support for domestic vaccine passports (*β*_wave1_ = 0.19, *p* = 0.000; *β*_wave2_ = 0.23, *p* = 0.000; *β*_wave3_ = 0.25, *p* = 0.000) is comprised of two indirect effects: utilitarian reasoning (*β*_wave1_ = 0.12, 95% CI [0.05, 0.19]) and anger (*β*_wave1_ = 0.03, 95% CI [0.01, 0.06]). The positive coefficient of anger is a product of two negative effects: anger is predicted by individualising foundations (*β*_wave1_ =  − 0.18, *p* = 0.000), and support for domestic passports is negatively predicted by anger (*β*_wave1_ =  − 0.16, *p* = 0.000). As seen in the total effect coefficients, the relationships strengthen across waves and are reflected in the indirect effects over time: anger (*β*_wave2_ = 0.05, 95% CI [0.02, 0.09]; *β*_wave3_ = 0.05, 95% CI [0.02, 0.09]), utilitarian reasoning (*β*_wave2_ = 0.10, 95% CI [0.03, 0.17]; *β*_wave3_ = 0.14, 95% CI [0.08, 0.21]) as well as the indirect effect of deontological reasoning in waves 2 and 3 (*β*_wave2_ = 0.04, 95% CI [0.00, 0.08]; *β*_wave3_ = 0.04, 95% CI [0.01, 0.08]). These results do not provide support for Hypothesis 1, where we stipulated individualising foundations would have a negative effect on support. However, we find some support for Hypotheses 3 and clarification on the mediating effect of reasoning as individualising foundations positively predict support mediated via its negative effect on the emotional reaction of anger (Hypothesis 3) and the positive effect of cognitive judgements.

We find no direct or total effect for the binding foundations. However, our model reports a total negative effect of the liberty foundation (*β*_wave1_ =  − 0.14, *p* = 0.007; *β*_wave2_ =  − 0.15, *p* = 0.007; *β*_wave3_ =  − 0.17, *p* = 0.002) on support for domestic vaccine passports. This total effect can be broken into two mediated paths: utilitarian reasoning and anger. The indirect effect of utilitarian reasoning is consistent over the 3 months (*β*_wave1_ =  − 0.10, 95% CI [− 0.17, − 0.02]; *β*_wave2_ =  − 0.09, 95% CI [− 0.16, − 0.02]; *β*_wave3_ =  − 0.13, 95% CI [− 0.21, − 0.05]). Similarly, the indirect negative effect of liberty on support for domestic vaccine passports, through anger also holds over time (*β*_wave1_ =  − 0.03, 95% CI [− 0.07, − 0.01]), *β*_wave2_ =  − 0.06, 95% CI [− 0.10, − 0.02]; *β*_wave3_ =  − 0.06, 95% CI [− 0.11, − 0.02]). These findings thus provide support for our hypothesis that liberty negatively predicts support (Hypothesis 2) and that the relationship is mediated by anger (Hypothesis 4) and support for cognitive arguments.

#### International Vaccine Passports

As with domestic vaccine passports, support for international vaccine passports was not directly predicted by either individualising, binding, or liberty foundations, but we find a total effects of individualising and liberty foundations.

The total effect relationships provide some support for Hypotheses 3 and for the mediating role of moral reasoning international vaccine passports. The total effect of individualising foundations on support is positive and significant (*β*_wave1_ = 0.19, *p* = 0.000; *β*_wave2_ = 0.19, *p* = 0.000; *β*_wave3_ = 0.22, *p* = 0.000), contrary to Hypothesis 1 for international passports. This is broken down into indirect effects of anger (*β*_wave1_ = 0.06, 95% CI [0.02, 0.10]; *β*_wave2_ = 0.03, 95% CI [0.01, 0.06]; *β*_wave3_ = 0.03, 95% CI [0.01, 0.06]), utilitarian reasoning (*β*_wave1_ = 0.11, 95% CI [0.05, 0.18]; *β*_wave2_ = 0.10, 95% CI [0.03, 0.18]; *β*_wave3_ = 0.17, 95% CI [0.10, 0.25]), and support for deontological reasoning in wave 3 (*β*_wave3_ = 0.04, 95% CI [0.01, 0.08]).

Binding foundations were not a significant direct predictor of support for international vaccine passports but had a significant total effect at waves 2 and 3 (*β*_wave2_ = 0.14, *p* = 0.013; *β*_wave3_ = 0.13, *p* = 0.015). The only indirect effect is through support of deontological reasoning at wave 2 (*β*_wave2_ = 0.04, 95% CI [0.00, 0.08]).

We further find consistent support for Hypotheses 2 and 4 where liberty has a consistent negative, total effect on support for international passports (*β*_wave1_ =  − 0.13, *p* = 0.013; *β*_wave2_ =  − 0.20, *p* = 0.000; *β*_wave3_ =  − 0.19 *p* = 0.000) with an indirect effect through anger (*β*_wave1_ =  − 0.07, 95% CI [− 0.12, − 0.03]; *β*_wave2_ =  − 0.05, 95% CI [− 0.09, − 0.02]; *β*_wave3_ =  − 0.03, 95% CI [− 0.06, − 0.01]). Utilitarian reasoning also mediated the relationship at the last two timepoints (*β*_wave2_ =  − 0.10, 95% CI [− 0.18, − 0.02]; *β*_wave3_ =  − 0.13, 95% CI [− 0.22, − 0.04]), providing additional support for the role of cognitive judgments.

### Longitudinal Analysis

To understand how support, anger and cognitive judgements changed over time, we conducted repeated measure ANOVAs to compare the mean scores over time. Following this, though not hypothesised a priori, we used a latent curve growth model to understand how moral foundations predicted changes in the mediators (emotional reactions, cognitive judgements) and the outcome variable (support for vaccine passports).

#### Domestic Vaccine Passports

Repeated measures ANOVA looking at changes in support for domestic vaccine passports over time shows a significant change in support from wave 2 to wave 3 *F*(1, 300) = 7.33, *p* = 0.007. Indeed, we find mean support increased over time (*M*_wave1_ = 4.24, SD_wave1_ = 1.79; *M*_wave2_ = 4.26, SD_wave2_ = 1.73; *M*_wave3_ = 4.41, SD_wave3_ = 1.70). Simultaneously, we find significant reductions in anger reactions from wave 2 to wave 3 *F*(1, 300) = 6.60, *p* = 0.011 with average mean scores decreasing (*M*_wave1_ = 2.39, SD_wave1_ = 1.63; *M*_wave2_ = 2.34, SD_wave2_ = 1.57; *M*_wave3_ = 2.22, SD_wave2_ = 1.52). Deontological reasoning shows no significant change over time, and utilitarian reasoning falls, just significantly from wave 1 to 2 *F*(1, 300) = 4.21, *p* = 0.041; *M*_wave1_ = 4.37, SD_wave1_ = 1.52; *M*_wave2_ = 4.31, SD_wave2_ = 1.48; *M*_wave3_ = 4.42, SD_wave3_ = 1.50).

To understand whether either of the moral foundation groupings: individualising (harm and fairness), binding (loyalty, purity, and authority) or liberty, predicted changes in support, we ran a latent curve growth model with the three moral foundation groups as predictors (see Beaujean, 2014, p. 85) and three mediators (emotional anger, utilitarian reasoning, deontological reasoning). Although we do not find any direct or indirect effects of moral foundations on changes in support for domestic passports, we find some marginal effects: the change (fall) in anger over time significantly predicts changes in support for domestic passports (*β* =  − 2.47, *p* = 0.045) and individualising foundations predict changes in both utilitarian reasoning (*β* = 0.29, *p* = 0.001) and deontological reasoning (*β* = 0.28, *p* = 0.002), albeit changes in the latter were not significant over time.

#### International Vaccine Passports

As with domestic passports, we ran repeated measure ANOVAs for changes in ratings of international vaccine passports. We find no significant change in support for international passports (*M*_wave1_ = 4.60, SD_wave1_ = 1.66; *M*_wave2_ = 4.60, SD_wave2_ = 1.62; *M*_wave3_ = 4.67, *SD*_wave3_ = 1.63), anger reactions, support for deontological reasoning, or support for utilitarian reasoning. We, therefore, do not analyse international passport data with a latent curve growth model as no change implies that cross-sectional analyses suffice.

### Robustness Checks

#### Domestic Vaccine Passports

In order to understand whether the effects were influenced by any covariates or demographics measured, we ran linear regressions for each time-point. We find that identifying as male (*β*_wave1_ = 0.11, *p* = 0.016), supporting the Labour party (*β*_wave1_ = 0.15, *p* = 0.034), being Christian (*β*_wave2_ = 0.16, *p* = 0.024), or Muslim (*β*_wave2_ = 0.14, *p* = 0.011), expressing concern for COVID (*β*_wave3_ = 0.11, *p* = 0.028), and, having more favourable attitudes towards vaccinations (*β*_wave1_ = 0.44, *p* = 0.000; *β*_wave2_ = 0.39, *p* = 0.000; *β*_wave3_ = 0.51, *p* = 0.000) positively predicted support for domestic passports at the specified timepoints. However, having recovered from COVID-19 (*β*_wave1_ =  − 0.10, *p* = 0.033) and high religiosity (i.e. valuing religion more highly) (*β*_wave2_ =  − 0.15, *p* = 0.033) negatively predicted support for passports. We separately measured religiosity and religious affiliation (e.g., identifying as Christian or Muslim) such that the latter asked individuals if they consider themselves associated with a particular religion, and the former asked how much they value religion as a whole, irrespective of their affiliation.

We run additional regressions to test the effect of our independent variables (moral foundations) and mediating constructs (anger, utilitarian reasoning, and deontological reasoning) when controlling for vaccine attitudes, finding vaccine attitudes only predict attitudes to a domestic vaccine passport in Wave 2 (*β*_wave1_ = 0.11, *p* = 0.003). Full tables can be found in Appendix 3.

#### International Vaccine Passports

Likewise, for international passports, we find that expressing concern for covid (*β*_wave3_ = 0.11, *p* = 0.035), identifying as male (*β*_wave1_ = 0.10, *p* = 0.026), being Hindu (*β*_wave2_ = 0.11, *p* = 0.032), Jewish (*β*_wave2_ = 0.10 *p* = 0.024), or Muslim (*β*_wave2_ = 0.13, *p* = 0.019) and, having more favourable attitudes towards vaccination (*β*_wave1_ = 0.43, *p* = 0.000; *β*_wave2_ = 0.40, *p* = 0.000; *β*_wave3_ = 0.48, *p* = 0.000) predicts higher support for international vaccine passports. We found no significant negative relationships, and the effect of attitude to vaccination only holds in Wave 2 (*β*_wave2_ = 0.10, *p* = 0.011) .

**Fig. 2:**
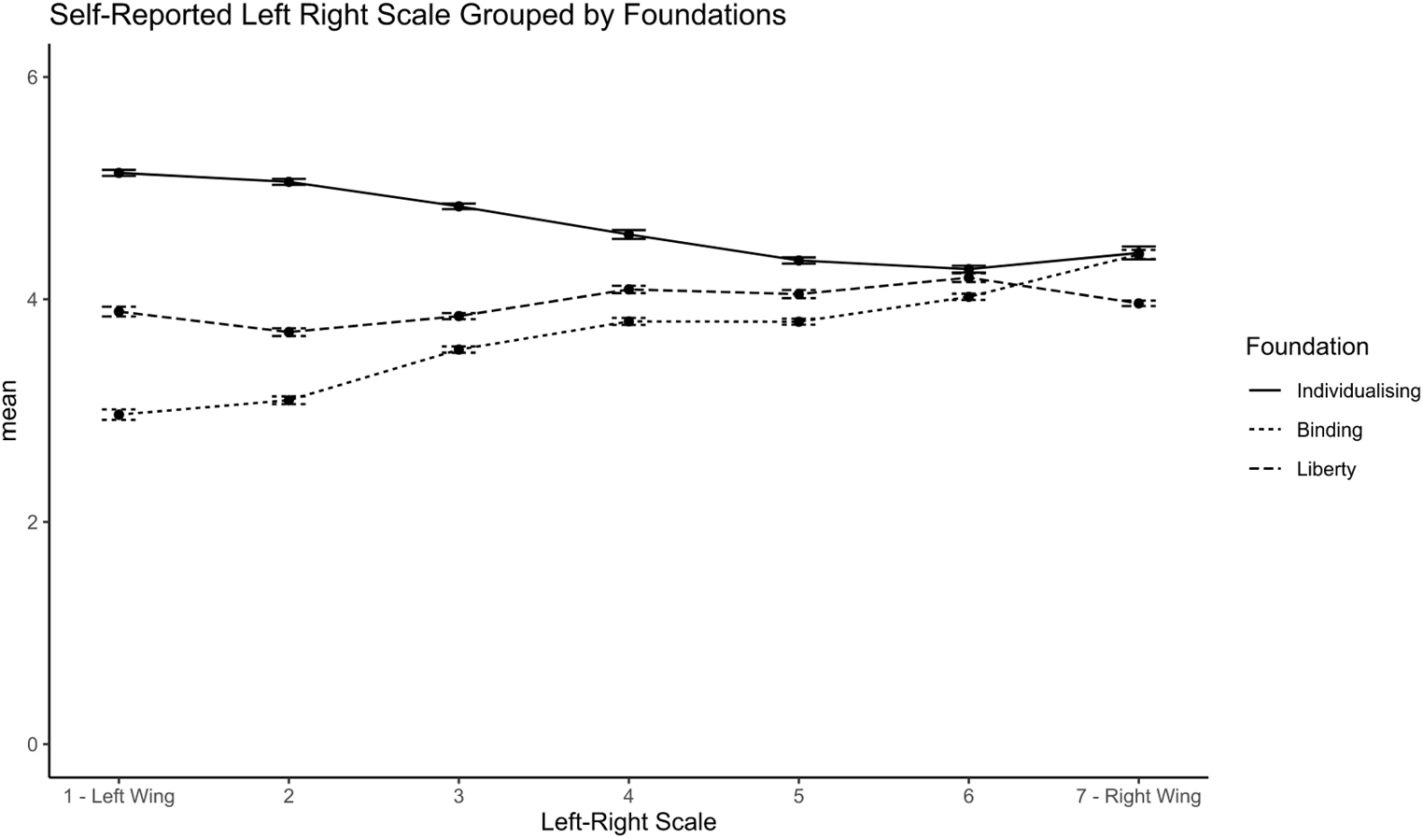
Self-reported political ideology by Moral Foundation Profile

**Fig. 3:**
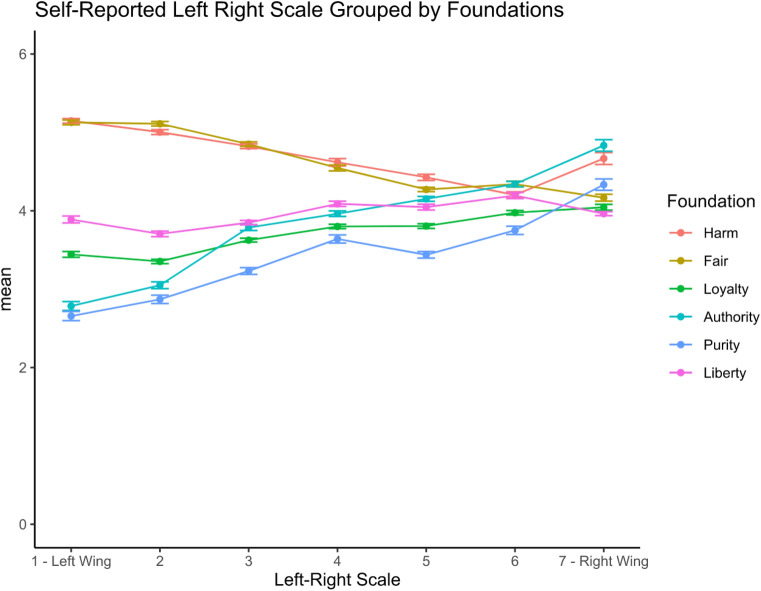
Self-reported political ideology by Moral Foundation

Figures 2, 3 below provide an overview of the relationship between political leaning and moral foundation profiles, as well as individual moral foundations.

## Discussion

### Theoretical Implications

The current research has made two novel contributions to the literature. For the first time, this paper investigates the moral roots predicting support for vaccine passports. Secondly, using moral foundations as a predictor of attitudes over time opens new avenues of research for future practitioners. We examine an understudied area. The existing body of literature investigating vaccine passports constitutes of attitude polling (Ibbetson, 2021), testing nudges to encourage acceptance (Sotis et al., 2021), its relationship to one’s willingness to receive the COVID-19 vaccine (de Figueiredo et al., 2021; Porat et al., 2021), and discussions of the legal, ethical, and practical considerations (Gostin & Hodge, 2020; Sleat et al., 2021; Wilford et al., 2021). Our study elucidates how the interplay of moral foundations and appraisals (cognitive and emotional) inform the formation of an opinion towards vaccine passports. This is an important research agenda, particularly as our research finds no effect of the binding foundations on support for vaccine passports, one of the more robust predictors of vaccination decisions (Amin et al., 2017), including actual COVID-19 vaccination rates (Reimer et al., 2022). This further highlights the need to study the moral reasoning towards vaccine passports separately from vaccination attitudes as they likely trigger different core moral values.

Contrary to Hypothesis 1, we find that the total effect of individualising foundations on support for both domestic and international passports is positively mediated by utilitarian reasoning and state anger. This relationship was stable across all three waves. We explore two possible reasons for this: (1) the directionality of the mediating constructs and (2) a media reporting bias. In line with Hypothesis 2, we find a consistently negative total effect of the liberty foundation. Together, the findings have implications for countries with low vaccination rates, those that have adopted the vaccine passport, and future uses of the vaccine passport.

Our initial prediction that individualising foundations would have a negative effect on support for vaccine passports was based on the measure’s potential to result in social harm and inequity. However, we observe that higher individualising foundations predicted more support for utilitarian arguments, which positively predicted support for vaccine passports. As such, our findings suggest that those with a relatively higher endorsement of the individualising foundations are more likely to use a form of utilitarian calculation grounded in principles of harm and fairness. It is thus possible that this type of moral reasoning involved elements such as rule utilitarianism and an interest in others’ welfare. Specifically, our findings point in the direction that individuals high in individualising foundations may have judged the rightness of the vaccine passport based on what would result in “the greatest good for the greatest number of people”, where “good” is defined as maximising collective benefit. This is  thus one possible explanation for why Hypothesis 1 was unsupported: individuals exhibit support for the vaccine passport as the measure can maximise the number of safe (uninfected) individuals in society and minimise harm with  concerns of harm (via potential infection) outweighed other fairness and harm concerns, such as inequality.

These decisions are likely tied in with other individual difference variables, such as one’s social value orientation, an individual’s preference for allocating resources in a proself (self-preference) and prosocial (other-regarding preference) manner (Pletzer et al., 2018), and one’s expectation of how much others will cooperate (Kukowski et al., 2021). Indeed, prior studies find that altruism (Murphy et al., 2021) and concern for others (Jung & Albarracin, 2021) positively predict vaccination. The observed positive effect of individualising foundations on support for vaccine passports may further elucidate the common predictors between vaccines and vaccine passports. Reimer et al. (2022) find that fairness endorsements predicted U.S. County level-vaccination rates.

In comparison, individualising foundations negatively predicted the second mediator (anger), which also negatively predicted support for vaccine passports. Given the prior work on emotional reactions to moral violations (Landmann & Hess, 2018), one expects anger to be elicited if a violation has taken place. Therefore, the lowered anger suggested that those holding individualising foundations did not feel violated by the implementation of vaccine passport policies. From the lens of a  collective action problem, this increased support due to lower anger lends credence  to the argument that higher anger has the potential to impede collective action (Brady & Crockett, 2019). Our results may also be indicative of a negativity bias (Soroka et al., 2019) in media reporting, which amplified perceived grievances against the measure on which our initial hypothesis was based. In media coverage, concerns of harm and fairness dominated the critique against vaccine passports (Cave et al., 2021), with concerns around discrimination, privacy, and inequity. Instead, our results suggest that endorsement of the individualising foundations is associated with the utilitarian reasoning that the benefits of the vaccine passport offset the potential harm to the unvaccinated minority.

Political liberals (individualising foundations), compared to political conservatives (binding foundation), have been found to have a lower need for order, structure, and higher openness to experience (Jost et al., 2003); thus, those with individualising morals should be more open to social change (Graham et al., 2013). Our results echo this line of thinking. Although this study did not find any effect of the binding foundations (loyalty, authority, purity), Bruchmann and LaPierre (2021) find that conservatives in the U.S. perceive violations of COVID-19 guidelines as more morally permissible than liberals, a difference that can be accounted for by their endorsement of the binding and individualising foundations, respectively. We find no effect of loyalty, authority, or purity (see Appendix 5) on support for domestic vaccine passports. However, despite an observed positive relationship between moral loyalty and vaccination rates in the U.S (Reimer et al., 2022), we find a negative relationship with loyalty in wave 2 on international vaccine passports and a positive relationship with authority in waves 2 and 3 (see Appendix 5). The ostensible differences between the moral bases of vaccination attitudes (or behaviour) and support for vaccine passports further motivate this study and highlights the necessity for an independent study of vaccine passports.

As predicted, we did find significant indirect negative relationships between the endorsement of the liberty foundation and support for domestic passports mediated by anger and utilitarian reasoning. This provided support for Hypothesis 4 and some clarity on the mediating role of cognitive judgments. The effect is consistent over time and is compatible with media reporting (Cave et al., 2021; Grossin et al., 2021). The endorsement of liberty values negatively predicted utilitarian reasoning, suggesting either that participants endorsing higher liberty values do not reason using the rule utilitarianism metric discussed above, or that they fundamentally disagreed with the idea that vaccine passports may benefit a greater proportion of society. Indeed, it is conceivable that those of a libertarian moral profile may define maximising “good” or collective benefit as maximising liberties. Thus lower support stems from the vaccine passports’ infringement on some liberties. We did not find the deontological statements, which were grounded in issues of liberty, to be a positive predictor (see Appendix 2 for full measure).

Liberty’s negative effect on support is also mediated by anger reactions. Given that individuals react to the violation of moral foundations with “moral outrage” (Brady et al., 2020) or a general “moral upset” (Atari et al., 2020), and that in addition to anger, disgust is also another common moral emotion that can contribute to the feeling of outrage, it is possible that although the news media tended to focus almost exclusively on anger, disgust might also play a role in shaping people’s reaction toward vaccine passports. A supplementary analysis was conducted to explore this possibility (see Appendix 4). Although the aggregated score of disgust and anger negatively predicted support, this effect is driven mainly by anger (see Appendix 4). This could also be interpreted as a form of psychological reactance aroused by experienced threats to freedom or liberty (Brehm & Brehm, 1981). Reactance is often captured as a mixture of anger and general negative sentiment (Brehm & Brehm, 1981; Quick & Stephenson, 2007) and has shown a previous association with non-compliance to COVID-19 measures (Díaz & Cova, 2022). In short, anger is a unique moral emotion in response to violating moral foundation in the context of vaccine passports.

Individualising and liberty foundations positively and negatively predict support for international passports, respectively. Both moral profiles indirectly affect anger and utilitarian appraisals across all three waves. Moral foundations are, therefore, a useful predictor of attitudes to both vaccine passports. We may apply the policy and business implications outlined in the next section to international and domestic passports. It is important to note that mean support for international passports was higher at baseline (wave 1) and did not significantly change over time.

This three-wave study is also unique in exploring moral foundations as a predictor of attitudes over time. It examines whether cross-sectional effects changed over time using higher-order moral foundations to predict attitude change. We present two key takeaways. Primarily, increases in support for domestic passports between May and July 2021 coincide with decreases in anger. Further analysis found that the downward change in anger reactions (mediator) predicted the upward trend in support for domestic passports (outcome). Although it is unclear what caused changes in anger, prior research has found that moral anger—relative to moral disgust—is a more flexible emotion (Russell & Giner-Sorolla, 2011). In this study, anger is a driver of support. However, the recent literature investigating the conjoint effect of anger and disgust as “moral outrage” (Brady et al., 2020) shows that changes in both emotions, predicted by moral values, can also inform communication over time.

Secondly, we find that endorsement of individualising foundations negatively predicted changes in reasoning (utilitarian and deontological appraisals), in line with the cross-sectional analysis where utilitarian reasoning is a positive mediator of individualising foundations and support. To our knowledge, no research has modelled moral foundations as a trait predictor for attitude changes over time. Despite being exploratory, we would contend that establishing moral foundations as a trait variable and investigating its predictive power over time are fruitful lines of research.

These findings thus provide initial evidence that moral foundations are helpful for predicting attitudes towards vaccine passports and have special implications for countries that are still having to rely on non-pharmaceutical interventions, due to lower vaccination rates, for example. In sub-Saharan Africa, some countries have vaccination rates as low as 3% at the time of writing (BBC The Visual & Data Journalism Team, 2022) and vaccine hesitancy is not uncommon (Adepoju, 2021; Menezes et al., 2021). This potentially lengthens the period in which both existing and new vaccine passports (Ledy, 2021; Pieterse, 2022) will be in use. However, the relevance is not limited to vaccine passports during COVID-19 or to the developing world. As new variants of COVID-19 continue to develop (WHO, 2022), and the prospect of future pandemics loom (Heymann et al., 2022; Penn, 2021; Smitham & Glassman, 2021), non-pharmaceutical interventions will remain relevant for the near future, as will research into their communication and policy design. At present, some states in the U.S. are introducing a national-level vaccine passport in conjunction with large private corporations (Kelleher, 2022), and regions such as Hong Kong only experienced a widespread outbreak of the virus at the beginning of 2022 (Master & Siu, 2022), introduced vaccine passports in February (Reuters, 2022). However, as noted in the limitations section, we would strongly call for the extension and replication of our findings.

### Policy and Business Implications

The relevance of our findings is twofold as it would not only inform the design of interventions to boost support for vaccine passports but can also serve to guide policy and business message framing. In the context of vaccine passports, the results imply that communication of the vaccine passports should prioritise liberty concerns to promote support. As Iyer et al. (2012) note, libertarians follow a distinct moral psychology that is not easily categorised into liberal or conservative. We measure political attitudes using the left–right scale, commonly used in the United Kingdom (Fieldhouse et al., 2019; Park et al., 2013). The results show that the endorsement of individualising foundations appears highest amongst those who endorse left-wing political orientation more strongly, whereas binding foundations are associated with a stronger endorsement of right-wing political orientation. The liberty foundation is not related to traditional left–right political orientation (see Figs. 2 and 3).

This may explain why liberty has been cited as a key reason to oppose COVID-19 measures across the political spectrum (Crawford, 2021; Özdüzen et al., 2021). We interpret this as evidence for the distinctiveness of a political and moral ideology grounded in liberty values. An understanding of liberal values can supplement other research findings that COVID-19 debates are not fractured along political lines (Jain et al., 2022). The finding that liberty is a negative predictor also appears to be a commonality with studies investigating moral bases of attitudes towards other pandemic countermeasures, including vaccines (Amin et al., 2017; Betsch & Böhm, 2018) and mask-wearing (He et al., 2021; Kaplan et al., 2021; Lehmann & Lehmann, 2021). This may be because such measures are seen to violate civil liberties and, as such, elicit a transgressive reaction (e.g., outrage) to violations. Indeed, earlier research finds that the efficacy of a messaging intervention aiming to increase compliance with COVID-19 measures depends on the individual endorsement of liberty values (Bokemper et al., 2022).

There are several ways to appeal to liberty values in communication, most commonly through moral reframing (Feinberg & Willer, 2019), which has shown demonstrable success as a political persuasion tool (Andrews et al., 2017; Feinberg & Willer, 2019; Hoover et al., 2018). Moral reframing is a “technique in which a position an individual would not normally support is reframed in a way that is consistent with that individual’s moral values” (Feinberg & Willer, 2019). Intuitively, the idea is a congruence between framing an issue, and moral values renders it more convincing. Prior studies have also used combinations of foundations to increase messaging efficacy (Wolsko, 2017). This is one of the least restrictive means of improving the uptake of such policy, which has been put forward as a guiding ethical principle in designing policy pertaining to vaccine passports (Thornton et al., 2022). Policy communicators should note, however, that studies examining the effect of the vaccine passport on vaccination attitudes have found null results (de Figueiredo et al., 2021; Sotis et al., 2021). Developing this line of thinking and teasing out the efficacy of interventions is vital, particularly in preparation for future pandemics. It is not always necessary that if a moral value predicts beliefs, its matched framing will increase compliance. For example, though Kaplan et al. (2021) find that the liberty value negatively predicts attitudes towards mask-wearing, liberty framing did not change belief or behaviour. In fact, the study finds that loyalty frames (also part of the higher-order binding structure) were more effective, highlighting two gaps future researchers may investigate.

Primarily, there is a notable need to establish the underlying moral values of attitudes and the efficacy of the corresponding message frame and its potential to influence behaviours. Vaccine hesitancy is predicted by liberty foundations (Amin et al., 2017), but message framing that highlights prosocial aspects (moral harm) is effective in increasing uptake (Jung & Albarracin, 2021) and higher loyalty predicted higher vaccination rates (Reimer et al., 2022). Conversely, the binding foundations (including loyalty) also predict finding transgressions of COVID-19 guidelines as more morally permissible (Bruchmann & LaPierre, 2021). This leads to the second gap; understanding the precise role of moral values and eliciting emotions in attitudes, collective behaviour, and policymaking. In this study, we find the prospect of vaccine passports elicits moral anger, which may play out in collective action, impeding it (Brady & Crockett, 2019) or perhaps as a motivator (Spring et al., 2018). Further research into behavioural outcomes is required to ascertain the impact of our findings on behaviour.

Thus, although the study of communication framing, informed by behavioural and social sciences, has been called upon to inform policy (Rimal & Lapinski, 2009; van Bavel et al., 2020), the direct application of results gleaned from online experiments are necessary but not sufficient to inform policy. As IJzerman et al. (2020) note, candidate measures should be tested in a systematic manner before their implementation. As such processes require the development of measures over time, a luxury that was not granted during COVID-19, this study on vaccine passports contributes to the testing of moral values and precedes tests of moral framing, in line with taking a preventative rather than reactionary approach to future pandemics (The Lancet Respiratory Medicine, 2022). Alternative interventions can draw on findings here testing messaging tailoring to sub-populations via morality matching or moral reframing, such as the finding that status quo and peer effect nudges can boost support for international passports (Sotis et al., 2021). The importance of value-matching messaging is arguably higher for attitudes towards domestic passports as opposed to international, given support is significantly lower at baseline.

The principle of moral reframing may also be applied in a business context, given many firms now bear the onus of setting a vaccine passport policy, particularly in the United States. Our surveys contribute to this effort in several ways: primarily, we find that discrimination concerns (part of the deontological appraisals) were not a primary justification for support or opposition to vaccine passports. Thus, although such concerns were featured in the media, they may not be the primary barrier to adopting a vaccine passport measure. Concerns of harm and fairness were found to be positively associated with support, initial evidence that though some are apprehensive about potential discrimination or liberty violations, avoiding further harm of COVID-19 to businesses and public health outweighed such concerns. Thus, the priority of communication should be to ease liberty concerns. Research has shown that followers’ sensitivity to the violation of certain foundations in the workplace is imbibed into moral perceptions of leaders (Weaver et al., 2014). Firms may thus incorporate this finding into their message framing of vaccine passports. Specifically, given individualising and binding foundations were not found to have negative relationships with support for vaccine passports, framing may generally address liberty concerns that the passport may provoke. For example, Kaplan et al. (2021) tested the effect of addressing liberty concerns for mask-wearing in which they note that mask-wearing can prevent more extreme limits on freedom (USC BCI, 2020). Similarly, those communicating vaccine passports may appeal to the idea that vaccine passports actually aim to promote access to liberties as they are conceived as an alternative to lockdowns (Satria et al., 2021), a more extreme restriction of freedoms. However, such messaging needs explicit testing prior to implementation. Studies may draw on resources such as vignettes of moral foundation violations developed and tested by Ekici et al. (2021), which include COVID-19 prompts that can be used to test sensitivity to moral foundation violations. Such resources would be useful in the design, testing, and implementation of interventions.

It is also important to note that our findings have implications for both domestic movement and international travel. Companies requiring employee travel may need to incorporate additional considerations into their communication strategy as the amount of travel required by a company might moderate the findings. Though this was not measured in our study, future studies may consider adding this in.

We recognize that some countries in Europe have begun to downgrade the risk of COVID-19 (Delfs & Rogers, 2022; Gualtieri, 2022), and vaccine passports may only be an ad hoc measure in certain regions. Still, our findings are relevant in other areas; some states in the United States only released passports in March 2022, including those that were initially opposed to the idea (Leonard, 2022). It is also common for the use of a passport for employees to be left at a company’s discretion in the United States (Withers, 2021). Beyond the ongoing COVID-19 pandemic, predicting support for vaccine passports is useful for understanding attitudes to similar measures in future health crises and pandemics.

### Limitations and Future Research Directions

Our study is naturally limited by several factors. First, we assume that moral foundations are stable over time. We also cannot disentangle the effects of exogenous variables over time in our study, which coincided with the vaccine drive in the U.K. and the emergence of the delta variant of COVID-19. There may have also been within-sample variation that affected our results as Scotland, Wales, and Northern Ireland (Department of Health Northern Ireland, 2021; Esson & Iredje, 2022; Lugonja, 2021) set their own vaccine passport mandates, independent of England. Our sample was limited to the U.K. population, a decision that was taken to conduct repeated sampling over a rapidly evolving policy. Though the sample was nationally representative, the findings would greatly benefit from testing on other populations and a larger sample.

Future research may consider extending our findings by evaluating the effectiveness of messaging framing the stability of moral foundations. Although we measure utilitarian and deontological reasoning, others may consider understanding liberty concerns at a more granular level, investigating which aspects of a measure violates the liberty foundation, including concerns about data privacy, transparency, or excessive government intervention, for example. This may help improve the identification of which other measures may be unsupported due to liberty violations. Similarly, we urge future studies to include the liberty foundation, given its distinctiveness as a moral-political profile. Moreover, as our study was in the U.K. and the libertarian movement is traditionally American (Kukathas, 2001), it is unclear precisely what the demarcations of liberal, conservative, and libertarian profiles would be in other countries. Although the profiles of individualising and binding have been held cross-culturally, including in the U.K. (Graham et al., 2013), we recognise that this is an atypical interpretation of the country’s political landscape, implying future research should explore the strength of this moral-political mapping and test the efficacy of moral reframing in other cultures. Magrath and Nichter (2022) recently demonstrated its potential success in the Indonesian context, albeit not by using moral foundations. Despite the MFQ-30 (the scale used here) being developed as a broad-brush moral theory drawing on anthropological findings across the globe (Graham et al., 2013), a new scale, MFQ-2 (Atari et al., 2022), which was released after this study, has been adapted for samples beyond the WEIRD demographic (Henrich et al., 2010). We would strongly encourage practitioners and policymakers to replicate these with the new MFQ-2 scale, particularly when applying these findings to cultures outside the U.K. or WEIRD populations in general. Although it is important to further examine factors that influence support toward vaccine passports and to investigate the replicability of the effects of moral foundations, it is equally important to note that the data for this study were collected in the U.K. when the implementation of vaccine passports was being discussed in the U.K. parliament, during the height of the COVID-19 pandemic. As such, an exact replication of the contextual factors is difficult to achieve. Future research and replications should consider this contextual influence and account for the potential impacts of extraneous variable(s). Relatedly, this also means that the data here is rich and unique in capturing public opinions during these distinctive historical circumstances.

Furthermore, this study adhered to the higher-order factor structure historically employed to group moral foundations in Western populations (individualising, binding, and liberty). We corroborate this by running an exploratory factor analysis (Appendix 5) and finding that the structure changes slightly; however, even with this change, our aforementioned do not change in significance or direction (Appendix 6). Since the current study focuses on the moral foundations of vaccine passports, it does not account for cultural and organisational values that may moderate the results. For example, Gelfand et al. (2021) measure variation in cultural tightness and find that looser cultures, with weaker social norms, were estimated to have almost five times the number of cases noting that areas of tighter culture (e.g., South Korea, China) abide by rules more strictly. Future research can, therefore, explore whether the effect of moral foundations on support for vaccine passports is moderated by cultural tightness.

## Conclusion

This study examined the moral underpinnings of support toward vaccine passports, a relatively unique pandemic countermeasure introduced across countries at the height of the pandemic. Vaccine passports were introduced as an entry requirement at bars, restaurants, and workplaces and international travel, and its use was widespread in regions around the world until very recently (Heung et al., 2022). Like other pandemic measures, its implementation was not without question and moralization. Therefore, it is paramount for any communicator of the measure (e.g., government authority, businesses, institutions, international bodies) to understand the impact of moral judgements towards the measure. Our three-wave longitudinal study highlights the cognitive and emotional reactions to implementing vaccine passports, grounded in core individual moral values. We present important insights about the role of moral foundations as a trait predictor of longitudinal attitudes and reveal their potential roles in message framing. This paper opens new avenues of research in studying the moral-political roots of contemporary opinions.

## Data Availability

The data that support the findings of this study are openly available at https://osf.io/dp8mn/.

## Appendix 1: Demographic Breakdown of Sample and 2011 U.K. Consensus

**Table:**

Demographic	Variable	*n*	Sample percentage (%)	Census percentage (%)
Gender*	Female	160	51	51
	Male	151	49	49
Age	18–27	35	11	14
	28–37	54	17	13
	38–47	62	20	15
	48–57	57	18	13
	Over 58	103	33	25
Political leaning	1 = Strongly left wing	17	5	–
	2	50	16	–
	3	80	26	–
	4 = In the center	94	30	–
	5	43	14	–
	6	24	8	–
	7 = Strongly right wing	3	1	–
Political party	Conservative	86	28	–
	DUP	1	0	–
	Green	25	8	–
	Labour	117	38	–
	Liberal democrats	28	9	–
	Other	42	14	–
	SNP	12	4	–
Race	Asian/Asian British	30	10	8
	Black/African/Caribbean/Black British	14	5	3
	Mixed or multiple ethnic groups	12	4	2
	Other	6	2	1
	Prefer not to say	4	1	–
	White	245	79	85

*Participants had the option to specify “Other” or “Prefer not to say”. None of our participants chose these options

## Appendix 2: Measuring Attitudes, Emotions, Reasoning Towards Vaccine Passport

Vaccine passports have been proposed as a measure for the next step of lifting COVID-19 restrictions. This would likely take the form of a digital certificate/identification granted by the government to every citizen that receives a vaccine or has recently tested negative for the COVID-19 virus. Similar schemes have already been implemented in several countries around the globe and your government is likely considering it. A vaccine passport would allow the holder to move more freely within a city, state, country or even cross-countries as it would ensure that you already have the antibodies to COVID-19. Some, however, have expressed concern regarding its implementation.

### Additional Description for Domestic Passport

Based on the description about vaccine passports, please indicate your agreement to the following statements with respect to a vaccine passport for use domestically (i.e., a passport in order to visit hotels, concerts, sports stadiums, theatres and nightclubs).

### Additional Description for International Passport

Based on the description about vaccine passports, please indicate your agreement to the following statements with respect to a vaccine passport for use internationally (i.e. a passport to travel cross-countries).

39 Item scale, all items measured on a 6-point likert scale with 1 = Strongly Disagree and 7 = Strongly Agree unless indicated otherwise. Emotions were measured on a 6-point scale with 1 = Not at all to 6 = Very Much.

### Full Measure of Cognitive Judgement, Perceptions, Attitudes, and Support for Domestic and International Vaccine Passports

**Table:**

The use of a domestic [international] vaccine passport would be…		
1	Ethical	Perceived ethicality
2	Appropriate	Perceived ethicality
3	The right thing to do	Perceived ethicality
If the vaccine passport was implemented for domestic [international] use, I would feel…		
4	Angry	Anger
5	Irritated	Anger
6	Annoyed	Anger
7	Contemptuous	Contempt
8	Disdainful	Contempt
9	Scornful	Contempt
10	Disgusted	Disgust
11	Repulsed	Disgust
12	Distasteful	Disgust
13	Anxiousness	Anxiety
14	Agitation	Anxiety
15	Worried	Anxiety
16	Excited	Excitement
17	Optimistic	Excitement
18	Enthusiastic	Excitement
19	Relieved	Relief
20	Comforted	Relief
21	Reassured	Relief
If a vaccine passport was implemented for domestic [international] use, I believe it would…		
22	Benefit society overall	Utilitarian reasoning
23	Benefit minorities	Utilitarian reasoning
24	Benefit me	Utilitarian reasoning
25	Be a fair and just measure	Deontological reasoning
26	Be a discriminatory measure	Deontological reasoning
27	Restrict personal freedoms	Deontological reasoning
28	Violate personal privacy	Deontological reasoning
29	Restrict social liberties	Deontological reasoning
30	Help drive business recovery	Utilitarian reasoning
31	Help drive economic recovery	Utilitarian reasoning
32	Be an effective public health measure	Utilitarian reasoning
33	Be effective in the fight against COVID-19	Utilitarian reasoning
What is your attitude toward the use of vaccine passport domestically [internationally]?		
34	Good–bad (6-point scale with 2 anchors)	Attitude
35	Positive–negative (6-point scale with 2 anchors)	Attitude
36	Favourable–unfavourable (6-point scale with 2 anchors)	Attitude
If a vaccine passport were to be implemented for domestic [international] use, I would…		
37	Support a government proposal for the passports	Support
38	Use the passport	Support
39	Support others using it	Support

The first wave survey also included the situated wise reasoning scale (Brienza et al., 2018), a 21-item measure designed to assess wisdom, or wise reasoning.

**Table:**

Measure	Wave 1	Wave 2	Wave 3
Domestic passports			
Support for vaccine passports	0.97	0.96	0.96
Deontological reasoning	0.96	0.96	0.95
Utilitarian reasoning	0.97	0.97	0.97
Anger	0.97	0.97	0.98
International passports			
Support for vaccine passports	0.96	0.96	0.95
Deontological reasoning	0.95	0.95	0.95
Utilitarian reasoning	0.96	0.96	0.97
Anger	0.97	0.97	0.98
Not passport-related			
Vaccine attitudes	0.97	0.97	0.95

## Appendix 3: Robustness Checks of Covariates

### Support for Domestic Vaccine Passports (All Covariates)

**Table:**

Predictors	Wave 1			Wave 2			Wave 3		
	Std. beta	Std. error	*p*	Std. beta	Std. error	*p*	Std. beta	Std. error	*p*
(Intercept)	− 0.000	0.043	**0.000**	− 0.000	0.044	**0.000**	0.000	0.044	**0.000**
Vaccine attitudes	0.440	0.088	**0.000**	0.392	0.083	**0.000**	0.506	0.080	**0.000**
Importance of religion	− 0.065	0.066	0.327	− 0.149	0.069	**0.033**	0.058	0.071	0.412
Political views*	0.097	0.063	0.123	0.065	0.066	0.322	0.073	0.067	0.278
Concern for COVID	0.070	0.048	0.149	0.065	0.050	0.191	0.108	0.049	**0.028**
Trust in science	0.079	0.082	0.340	0.062	0.089	0.484	0.063	0.089	0.479
Trust in vaccines	0.065	0.102	0.527	0.144	0.101	0.153	0.071	0.103	0.487
Trust in doctors	0.068	0.070	0.333	− 0.026	0.075	0.729	0.049	0.074	0.510
Age	0.038	0.069	0.584	− 0.082	0.065	0.208	− 0.019	0.056	0.732
Education	− 0.051	0.048	0.283	− 0.045	0.050	0.361	− 0.043	0.050	0.399
Household size	0.043	0.051	0.407	0.020	0.054	0.708	0.067	0.055	0.220
Income	− 0.049	0.048	0.308	− 0.074	0.050	0.139	− 0.047	0.051	0.351
Male = 1	0.110	0.045	**0.016**	0.075	0.047	0.113	0.035	0.047	0.450
White = 1	− 0.047	0.056	0.406	− 0.055	0.057	0.337	− 0.078	0.058	0.178
Vaccinated = Yes	− 0.043	0.067	0.525	0.096	0.064	0.136	− 0.008	0.060	0.893
Liberal democrats^+^ = 1	0.087	0.055	0.113	0.096	0.057	0.096	0.109	0.057	0.059
Conservative = 1	0.076	0.075	0.314	0.058	0.078	0.460	0.045	0.077	0.562
Labour = 1	0.152	0.071	**0.034**	0.051	0.075	0.501	0.080	0.075	0.281
Democratic Unionist Party = 1	− 0.009	0.044	0.834	0.005	0.046	0.916	0.032	0.045	0.483
Scottish National Party = 1	− 0.019	0.049	0.708	0.018	0.052	0.738	− 0.022	0.052	0.668
Green Party = 1	0.093	0.056	0.097	0.029	0.059	0.623	0.087	0.058	0.135
Medically vulnerable at home = 1	− 0.002	0.048	0.964	0.030	0.051	0.557	0.005	0.050	0.920
Carer = 1	0.079	0.048	0.103	0.047	0.049	0.342	0.004	0.049	0.941
Recovered from COVID = 1	− 0.102	0.047	**0.033**	− 0.062	0.048	0.202	− 0.010	0.049	0.830
Recovered from long COVID = 1	0.019	0.047	0.695	0.039	0.049	0.419	0.031	0.048	0.523
Christian = 1	0.072	0.065	0.270	0.157	0.069	**0.024**	0.015	0.069	0.827
Buddhist = 1	0.081	0.044	0.069	0.066	0.046	0.150	0.050	0.045	0.275
Hindu = 1	0.044	0.049	0.369	0.088	0.052	0.092	0.028	0.052	0.597
Jewish = 1	0.082	0.044	0.067	0.060	0.046	0.192	0.070	0.046	0.126
Muslim = 1	0.037	0.053	0.480	0.142	0.056	**0.011**	− 0.021	0.055	0.702
Sikh = 1	0.019	0.048	0.689	0.024	0.051	0.641	− 0.040	0.050	0.425
Other religion = 1	− 0.024	0.046	0.609	− 0.027	0.049	0.576	− 0.058	0.049	0.236
Observations	349			328			311		
*R*^2^/*R*^2^ adjusted	0.415/0.358			0.413/0.352			0.457/0.397		

Bold values indicate significance *p* value less than 0.05

*Higher number indicates more right-wing

### Support for Domestic Vaccine Passports (Higher-Order Foundations)

**Table:**

Predictors	Wave 1			Wave 2			Wave 3		
	Std. beta	Std. error	*p*	Std. beta	Std. error	*p*	Std. beta	Std. error	*p*
(Intercept)	− 0.000	0.028	0.164	− 0.000	0.029	**0.003**	− 0.000	0.025	0.065
Vaccine attitude	0.056	0.038	0.139	0.110	0.037	**0.003**	0.032	0.035	0.360
Binding	0.006	0.029	0.847	− 0.025	0.030	0.412	− 0.005	0.026	0.849
Individualising	0.031	0.029	0.279	0.043	0.030	0.150	0.017	0.026	0.517
Liberty	0.014	0.029	0.644	0.041	0.031	0.183	0.045	0.026	0.089
Anger	− 0.140	0.051	**0.007**	− 0.195	0.053	**0.000**	− 0.250	0.046	**0.000**
Deontological reasoning	0.327	0.036	**0.000**	0.294	0.038	**0.000**	0.315	0.033	**0.000**
Utilitarian reasoning	0.616	0.044	**0.000**	0.539	0.046	**0.000**	0.586	0.042	**0.000**
Observations	349			328			311		
*R*^2^/*R*^2^ adjusted	0.734/0.728			0.731/0.725			0.810/0.806		

Bold values indicate significance *p* value less than 0.05

### Support for International Vaccine Passports

**Table:**

Predictors	Wave 1			Wave 2			Wave 3		
	Std. beta	Std. error	*p*	Std. beta	Std. error	*p*	Std. beta	Std. error	*p*
(Intercept)	0.000	0.043	**0.000**	0.000	0.043	**0.000**	− 0.000	0.047	**0.000**
Vaccine attitudes	0.428	0.088	**0.000**	0.395	0.081	**0.000**	0.480	0.085	**0.000**
Importance of religion	0.005	0.066	0.940	− 0.070	0.067	0.303	0.047	0.075	0.535
Political views*	0.107	0.062	0.088	0.096	0.064	0.135	0.073	0.072	0.310
Concern for COVID	0.079	0.048	0.103	0.031	0.048	0.524	0.110	0.052	**0.035**
Trust in science	0.059	0.082	0.469	0.058	0.087	0.501	0.118	0.094	0.209
Trust in vaccines	0.108	0.102	0.288	0.103	0.098	0.292	0.028	0.109	0.798
Trust in doctors	0.011	0.069	0.879	0.055	0.072	0.447	− 0.055	0.078	0.481
Age	− 0.086	0.068	0.209	− 0.104	0.063	0.100	− 0.052	0.060	0.390
Education	− 0.014	0.047	0.763	− 0.020	0.048	0.681	− 0.048	0.054	0.368
Household size	0.016	0.051	0.749	− 0.023	0.053	0.665	0.009	0.058	0.873
Income	− 0.046	0.048	0.335	− 0.049	0.049	0.320	0.017	0.054	0.751
Male = 1	0.100	0.045	**0.026**	0.043	0.046	0.348	0.048	0.050	0.336
White = 1	− 0.025	0.056	0.653	0.015	0.056	0.783	− 0.009	0.062	0.881
Vaccinated = Yes	0.097	0.067	0.146	0.118	0.062	0.059	0.028	0.064	0.667
Liberal democrats = 1	0.076	0.055	0.166	0.085	0.056	0.131	0.051	0.061	0.400
Conservative = 1	0.047	0.075	0.533	0.085	0.076	0.263	0.076	0.082	0.353
Labour = 1	0.126	0.071	0.077	0.114	0.073	0.117	0.113	0.079	0.154
DUP = 1	0.033	0.044	0.448	0.016	0.044	0.716	0.043	0.048	0.377
SNP = 1	0.035	0.049	0.472	0.011	0.051	0.828	− 0.012	0.055	0.835
Green Party = 1	0.036	0.055	0.511	0.046	0.057	0.417	0.027	0.062	0.660
Medically vulnerable at home = 1	− 0.023	0.048	0.635	0.002	0.049	0.974	− 0.031	0.053	0.555
Carer = 1	0.084	0.048	0.081	0.074	0.048	0.121	0.038	0.052	0.460
Recovered from COVID = 1	− 0.060	0.047	0.205	− 0.090	0.047	0.057	− 0.052	0.052	0.319
Recovered from long COVID = 1	− 0.004	0.047	0.934	0.049	0.047	0.305	0.044	0.051	0.394
Christian = 1	0.022	0.065	0.741	0.128	0.067	0.058	− 0.004	0.073	0.952
Buddhist = 1	0.048	0.044	0.277	0.071	0.044	0.110	0.031	0.048	0.525
Hindu = 1	0.052	0.049	0.290	0.108	0.050	**0.032**	0.067	0.055	0.226
Jewish = 1	0.083	0.044	0.062	0.102	0.045	**0.024**	− 0.042	0.049	0.386
Muslim = 1	0.068	0.052	0.192	0.128	0.054	**0.019**	0.046	0.059	0.432
Sikh = 1	0.034	0.048	0.479	0.085	0.049	0.083	− 0.005	0.053	0.923
Other religion = 1	− 0.010	0.046	0.827	− 0.028	0.047	0.559	− 0.037	0.052	0.480
Observations	349			328			311		
*R*^2^/*R*^2^ adjusted	0.422/0.365			0.447/0.389			0.386/0.318		

Bold values indicate significance *p* value less than 0.05

### Support for International Passports

**Table:**

Predictors	Wave 1			Wave 2			Wave 3		
	Std. beta	Std. error	*p*	Std. beta	Std. error	*p*	Std. beta	Std. error	*p*
(Intercept)	0.000	0.026	0.216	0.000	0.028	0.112	− 0.000	0.028	0.211
Vaccine attitude	0.018	0.036	0.624	0.096	0.038	**0.011**	− 0.040	0.040	0.315
Binding	− 0.011	0.027	0.693	0.044	0.030	0.142	0.032	0.029	0.274
Individualising	0.012	0.027	0.647	0.033	0.029	0.256	− 0.017	0.029	0.552
Liberty	0.037	0.027	0.181	− 0.009	0.030	0.764	− 0.022	0.029	0.458
Anger	− 0.286	0.048	**0.000**	− 0.196	0.046	**0.000**	− 0.162	0.047	**0.001**
Deontological reasoning	0.204	0.032	**0.000**	0.272	0.037	**0.000**	0.304	0.034	**0.000**
Utilitarian reasoning	0.594	0.040	**0.000**	0.561	0.044	**0.000**	0.706	0.044	**0.000**
Observations	349			328			311		
*R*^2^/*R*^2^ adjusted	0.774/0.769			0.741/0.736			0.767/0.762		

Bold values indicate significance *p* value less than 0.05

## Appendix 4: Robustness Checks for Moral Outrage

These regressions explore the potential effects of moral outrage (or anger & disgust, as well as separately). Please note disgust was not measured in Wave 3.

### Support for Domestic Passports (Moral Outrage–Composite Score)

**Table:**

Predictors	Wave 1			Wave 2		
	Std. beta	Std. error	p	Std. beta	Std. error	p
(Intercept)	− 0.000	0.028	0.189	− 0.000	0.029	**0.005**
Vaccine attitude	0.052	0.038	0.172	0.101	0.038	**0.009**
Binding	0.010	0.029	0.725	− 0.020	0.030	0.507
Individualising	0.036	0.029	0.211	0.050	0.030	0.100
Liberty	0.014	0.029	0.638	0.044	0.031	0.155
Moral outrage	− 0.146	0.050	**0.004**	− 0.184	0.053	**0.001**
Deontological reasoning	0.329	0.035	**0.000**	0.304	0.037	**0.000**
Utilitarian reasoning	0.614	0.043	**0.000**	0.553	0.045	**0.000**
Observations	349			328		
*R*^2^/*R*^2^ adjusted	0.735/0.729			0.730/0.724		

Bold values indicate significance *p* value less than 0.05

### Support for Domestic Passports (Disgust and Anger)

**Table:**

Predictors	Wave 1			Wave 2		
	Std. beta	Std. error	*p*	Std. beta	Std. error	*p*
(Intercept)	0.000	0.028	0.192	− 0.000	0.029	**0.004**
Vaccine Attitude	0.051	0.038	0.183	0.106	0.038	**0.006**
Binding	0.009	0.029	0.761	− 0.024	0.030	0.433
Individualising	0.034	0.029	0.236	0.045	0.030	0.140
Liberty	0.014	0.029	0.624	0.043	0.031	0.168
Disgust	− 0.057	0.050	0.253	− 0.028	0.059	0.639
Anger	− 0.101	0.061	0.100	− 0.176	0.068	**0.010**
Deontological Reasoning	0.325	0.036	**0.000**	0.293	0.038	**0.000**
Utilitarian reasoning	0.610	0.044	**0.000**	0.538	0.046	**0.000**
Observations	349			328		
*R*^2^/*R*^2^ adjusted	0.735/0.729			0.731/0.725		

Bold values indicate significance *p* value less than 0.05

### Support for International Passports (Moral Outrage–Composite Score)

**Table:**

Predictors	Wave 1			Wave 2		
	Std. beta	Std. error	*p*	Std. beta	Std. error	*p*
(Intercept)	0.000	0.026	0.211	0.000	0.029	0.222
Vaccine attitude	0.020	0.036	0.585	0.080	0.041	0.052
Binding	0.001	0.027	0.960	0.043	0.030	0.158
Individualising	0.020	0.027	0.456	0.031	0.030	0.299
Liberty	0.036	0.027	0.195	− 0.011	0.031	0.722
Moral outrage	− 0.269	0.046	**0.000**	− 0.121	0.047	**0.011**
Deontological reasoning	0.216	0.031	**0.000**	0.307	0.036	**0.000**
Utilitarian reasoning	0.605	0.039	**0.000**	0.626	0.040	**0.000**
Observations	349			328		
*R*^2^/*R*^2^ adjusted	0.773/0.768			0.732/0.726		

Bold values indicate significance *p* value less than 0.05

### Support for International Passports (Disgust and Anger)

**Table:**

Predictors	Wave 1			Wave 2		
	Std. beta	Std. error	*p*	Std. beta	Std. error	*p*
(Intercept)	0.000	0.026	0.240	0.000	0.028	0.153
Vaccine attitude	0.013	0.036	0.713	0.082	0.039	**0.035**
Binding	− 0.006	0.027	0.829	0.043	0.030	0.147
Individualising	0.014	0.027	0.589	0.036	0.029	0.221
Liberty	0.038	0.027	0.167	− 0.004	0.030	0.886
Disgust	− 0.063	0.049	0.195	− 0.086	0.055	0.119
Anger	− 0.239	0.060	**0.000**	− 0.134	0.061	**0.029**
Deontological reasoning	0.203	0.032	**0.000**	0.271	0.036	**0.000**
Utilitarian reasoning	0.590	0.040	**0.000**	0.559	0.044	**0.000**
Observations	349			328		
*R*^2^/*R*^2^ adjusted	0.775/0.770			0.743/0.737		

Bold values indicate significance *p* value less than 0.05

## Appendix 5: Robustness Checks (Factor Structure of Moral Foundations)

### Support for Domestic Passports (Individual-Level Foundations)

**Table:**

Predictors	Wave 1			Wave 2			Wave 3		
	Std. beta	Std. error	*p*	Std. beta	Std. error	*p*	Std. beta	Std. error	*p*
(Intercept)	− 0.000	0.028	0.076	− 0.000	0.029	**0.004**	− 0.000	0.025	0.099
Vaccine attitude	0.057	0.038	0.136	0.116	0.038	**0.002**	0.031	0.035	0.384
Harm	0.012	0.038	0.750	0.001	0.040	0.983	0.026	0.034	0.444
Fairness	0.022	0.037	0.553	0.045	0.038	0.240	− 0.004	0.034	0.911
Loyalty	− 0.069	0.060	0.246	− 0.036	0.060	0.552	− 0.064	0.052	0.221
Authority	0.040	0.040	0.317	− 0.018	0.041	0.656	0.038	0.036	0.285
Purity	0.006	0.038	0.886	0.022	0.039	0.583	− 0.013	0.035	0.721
Liberty	0.060	0.052	0.247	0.064	0.053	0.225	0.088	0.046	0.057
Anger	− 0.137	0.052	**0.009**	− 0.192	0.054	**0.000**	− 0.249	0.046	**0.000**
Deontological reasoning	0.327	0.036	**0.000**	0.293	0.038	**0.000**	0.314	0.034	**0.000**
Utilitarian reasoning	0.618	0.044	**0.000**	0.538	0.046	**0.000**	0.588	0.042	**0.000**
Observations	349			328			311		
*R*^2^/*R*^2^ adjusted	0.735/0.727			0.732/0.723			0.812/0.805		

Bold values indicate significance *p* value less than 0.05

### Support for International Passports (Individual-Level Foundations)

**Table:**

Predictors	Wave 1			Wave 2			Wave 3		
	Std. beta	Std. error	*p*	Std. beta	Std. error	*p*	Std. beta	Std. error	*p*
(Intercept)	0.000	0.026	0.276	0.000	0.028	**0.008**	− 0.000	0.027	0.267
Vaccine attitude	0.016	0.036	0.657	0.095	0.037	**0.012**	− 0.050	0.040	0.212
Harm	0.010	0.035	0.783	0.002	0.038	0.960	0.019	0.038	0.623
Fairness	0.009	0.034	0.784	0.036	0.037	0.333	− 0.041	0.038	0.280
Loyalty	− 0.097	0.055	0.078	− 0.155	0.058	**0.008**	− 0.056	0.057	0.334
Authority	0.030	0.037	0.407	0.122	0.039	**0.002**	0.105	0.039	**0.008**
Purity	0.011	0.035	0.757	0.026	0.038	0.494	− 0.047	0.038	0.225
Liberty	0.102	0.048	**0.033**	0.095	0.051	0.066	0.014	0.050	0.784
Anger	− 0.285	0.048	**0.000**	− 0.203	0.046	**0.000**	− 0.157	0.048	**0.001**
Deontological reasoning	0.203	0.032	**0.000**	0.256	0.036	**0.000**	0.308	0.034	**0.000**
Utilitarian reasoning	0.599	0.040	**0.000**	0.561	0.043	**0.000**	0.716	0.045	**0.000**
Observations	349			328			311		
*R*^2^/*R*^2^ adjusted	0.776/0.769			0.752/0.744			0.773/0.765		

Bold values indicate significance *p* value less than 0.05

We also ran an exploratory factor analysis on SPSS using all the items from the moral foundation scales (including liberty), with a requirement of 3 components. The factor loadings were as follows in the table below, with two liberty items loading onto the ‘individualising’ factor and the ‘binding’ factor. These are highlighted in italics. The Cronbach alphas for these higher order structures are individualising; with new liberty item (*α* = 0.83), binding; with new liberty item (*α* = 0.89) and liberty; without the 2 items (*α* = 0.70).mfq_lib_ec1 = Whether or not private property was respected.mfq_lib_lf1 = Whether or not everyone was free to do as they wanted.

**Table:**

Item	Binding	Individualising	Liberty
mfq_a4	**0.709**	− 0.092	0.054
mfq_a1	**0.703**	0.166	− 0.050
mfq_a2	**0.701**	0.051	− 0.178
mfq_d2	**0.689**	0.221	− 0.046
mfq_l1	**0.649**	0.168	− 0.016
mfq_d6	**0.618**	− 0.097	0.091
mfq_l4	**0.617**	− 0.274	0.074
mfq_d5	**0.604**	− 0.029	0.167
mfq_a3	**0.568**	0.379	− 0.118
mfq_d4	**0.553**	0.180	0.048
mfq_a5	**0.529**	− 0.205	0.073
mfq_d3	**0.528**	0.057	0.138
mfq_l2	**0.519**	0.368	− 0.061
mfq_a6	**0.508**	− 0.248	0.225
mfq_d1	**0.508**	0.371	− 0.087
*mfq_lib_ec1*	***0.488***	*0.299*	*0.032*
mfq_l6	**0.436**	− 0.028	0.036
mfq_l3	**0.434**	0.365	0.065
mfq_lib_ec2	0.420	− 0.156	**0.450**
mfq_l5	0.309	0.094	0.015
mfq_h2	0.220	**0.616**	− 0.123
mfq_f6	0.201	0.322	0.034
mfq_lib_ec6	0.155	0.079	**0.720**
mfq_lib_ec3	0.147	− 0.068	**0.628**
mfq_h3	0.128	**0.611**	− 0.228
mfq_h5	0.128	**0.421**	0.144
mfq_lib_ec4	0.108	− 0.111	**0.525**
mfq_h1	0.063	**0.671**	− 0.120
mfq_h4	0.062	**0.619**	0.179
mfq_f2	0.053	**0.649**	− 0.090
mfq_lib_ec5	0.035	− 0.244	**0.332**
*mfq_lib_lf1*	*0.030*	***0.355***	*0.329*
mfq_h6	0.024	**0.426**	0.127
mfq_f1	− 0.005	**0.749**	− − 0.065
mfq_f4	− 0.038	**0.537**	0.272
mfq_f3	− 0.067	**0.593**	− 0.021
mfq_lib_lf2	− 0.157	0.189	**0.704**
mfq_lib_lf3	− 0.186	0.243	**0.616**
mfq_f5	− 0.273	**0.517**	− 0.127

Bold values indicate significance *p* value less than 0.05

## Appendix 6: Re-analysis

After conducting the EFA described in Appendix 5, and creating the new higher-level variables for the individualising, binding, and liberty foundations, we re-ran our main path analyses over 3 waves. We find that all the results were confirmed (i.e., no changes in significance). We only find a slight change in the beta values of the following relationships on:

1. Support of domestic passports as the outcome variable:
  - Total effect of individualising foundations (*β*_wave3_ = 0.26, *p* = 0.000).
  - The total effect of liberty foundations (*β*_wave1_ =  − 0.16, *p* = 0.002) and the pathways of anger (*β*_wave1_ =  − 0.04, 95%CI [− 0.07, − 0.01], *β*_wave2_ =  − 0.05, 95% CI) and utilitarian reasoning (*β*_wave2_ =  − 0.08, 95%CI [− 0.16, − 0.01]; *β*_wave3_ =  − 0.11, 95%CI [− 0.19, − 0.03]).
2. Support of international passports as the outcome variable:
  - The total effect of individualising foundations (*β*_wave1_ = 0.20, *p* = 0.000).
  - The total effect of the liberty foundations (*β*_wave1_ =  − 0.15, *p* = 0.005) and the pathways of utilitarian reasoning (*β*_wave2_ =  − 0.08, 95% CI [− 0.17, − 0.01]; *β*_*wave3*_ =  − 0.12, 95% CI [− 0.21, − 0.03]).
  - The total effect of the binding foundations (*β*_wave3_ = 0.12, *p* = 0.028).

The observed relationships (first 2 bullet points) did not change in the longitudinal analysis in terms of significance but only slightly in terms of the coefficients. However, the last two bullet points were newly significant relationships.

- Change in anger (fall) significantly predicted changes in support for domestic passports (*β* =  − 2.47, *p* = 0.041).
- Individualising foundations predicted changes in utilitarian reasoning ((*β* = 0.28, *p* = 0.002) and deontological reasoning (*β* = 0.26, *p* = 0.003).
- Binding foundations also marginally predicted changes in deontological reasoning (*β* =  − 0.18, *p* = 0.048).
- Liberty foundations predicted changes in utilitarian reasoning (*β* =  − 0.19, *p* = 0.038).

## Footnotes

1 We relied on Prolific’s sampling tool to recruit a nationally representative sample. It is based on the 2011 census data using only data from England: https://researcher-help.prolific.co/hc/en-gb/articles/360019238413#heading-1. We compare our output to the census in Appendix 1.

2 One participant privately messaged us saying they misinterpreted our scaling system but were unable to change their answers.

3 Long-COVID refers to long-term effects of COVID-19 and covers individuals who continue to have the effects of the virus months after initially falling ill. Symptoms are wide-ranging and may include breathlessness, chronic fatigue, “brain fog”, anxiety and stress (Definition from the National Health Service Website in the UK).

## References

[CR1] Adepoju P (2021). Africa is waging a war on COVID anti-vaxxers. Nature Medicine.

[CR2] Albrecht D (2022). Vaccination, politics and COVID-19 impacts. BMC Public Health.

[CR3] Allegretti, A. (2021). Vaccine passport plan intended to coax young to have jabs, says Raab. *The Guardian*. Retrieved from https://www.theguardian.com/world/2021/jul/29/vaccine-passport-plan-intended-to-coax-young-to-have-jabs-says-raab

[CR4] Amin AB, Bednarczyk RA, Ray CE, Melchiori KJ, Graham J, Huntsinger JR, Omer SB (2017). Association of moral values with vaccine hesitancy. Nature Human Behaviour.

[CR5] Andrews AC, Clawson RA, Gramig BM, Raymond L (2017). Finding the right value: Framing effects on domain experts. Political Psychology.

[CR6] Asri A, Asri V, Renerte B, Föllmi-Heusi F, Leuppi JD, Muser J, Nüesch R, Schuler D, Fischbacher U (2021). Wearing a mask—For yourself or for others? Behavioral correlates of mask wearing among COVID-19 frontline workers. PLoS ONE.

[CR7] Associated Press. (2021). Moroccans protest vaccine pass required for work, travel. *Associated Press*. Retrieved from https://apnews.com/article/coronavirus-pandemic-lifestyle-travel-air-travel-north-africa-9a116e046ab9c0455d6e19c31e454b3c

[CR8] Associated Press. (2022). Vaccine passport protests in Europe draw thousands of people. *ABC News*. Retrieved from https://abcnews.go.com/Health/wireStory/vaccine-passport-protests-europe-draw-thousands-people-82415992

[CR9] Atari, M., Haidt, J., Graham, J., Koleva, S., Stevens, S. T., & Dehghani, M. (2022). *Morality beyond the WEIRD: How the nomological network of morality varies across cultures*. 10.31234/osf.io/q6c9r 10.1037/pspp0000470 37589704

[CR10] Atari M, Mostafazadeh Davani A, Dehghani M (2020). Body maps of moral concerns. Psychological Science.

[CR11] Baldner C, Pierro A (2019). Motivated prejudice: The effect of need for closure on anti-immigrant attitudes in the United States and Italy and the mediating role of binding moral foundations. International Journal of Intercultural Relations.

[CR12] Barceló J, Sheen GCH (2020). Voluntary adoption of social welfare-enhancing behavior: Mask-wearing in Spain during the COVID-19 outbreak. PLoS ONE.

[CR13] Baril GL, Wright JC (2012). Different types of moral cognition: Moral stages versus moral foundations. Personality and Individual Differences.

[CR14] Bavel JJ, Baicker K, Boggio PS, Capraro V, Cichocka A, Cikara M, Crockett MJ, Crum AJ, Douglas KM, Druckman JN, Drury J (2020). Using social and behavioural science to support COVID-19 pandemic response. Nature Human Behaviour.

[CR15] BBC News. (2021a). Covid-19: Vaccine passports could create “two-tier society”, equality watchdog warns. *BBC*. Retrieved from https://www.bbc.co.uk/news/uk-56755161

[CR16] BBC News. (2021b). Vaccine passports make me even more reluctant to get a Covid jab. *BBC.* Retrieved from https://www.bbc.co.uk/news/newsbeat-58505658

[CR17] BBC The Visual and Data Journalism Team. (2022). Covid vaccines: How fast is progress around the world? *BBC News*. Retrieved March 2, 2022, from https://www.bbc.co.uk/news/world-56237778

[CR18] Beaujean AA (2014). Latent variable modeling using R.

[CR19] Betsch C, Böhm R (2018). Moral values do not affect prosocial vaccination. Nature Human Behaviour.

[CR20] Betsch C, Böhm R, Chapman GB (2015). Using behavioral insights to increase vaccination policy effectiveness. Policy Insights from the Behavioral and Brain Sciences.

[CR300] Brienza JP, Kung FYH, Santos HC, Bobocel R, Grossman I (2018). Situated WIse Reasoning Scale (SWIS) [Database record]. APA PsycTests.

[CR21] Bokemper S, Huber G, James E, Gerber A, Omer S (2022). Testing persuasive messaging to encourage COVID-19 risk reduction. PLoS ONE.

[CR22] Boyle GJ (1984). Reliability and validity of Izard’s differential emotions scale. Personality and Individual Differences.

[CR23] Brady WJ, Crockett MJ (2019). How effective is online outrage?. Trends in Cognitive Sciences.

[CR24] Brady WJ, Crockett MJ, Van Bavel JJ (2020). The MAD model of moral contagion: The role of motivation, attention, and design in the spread of moralized content online. Perspectives on Psychological Science.

[CR25] Brehm SS, Brehm JW (1981). Psychological reactance: A theory of freedom and control.

[CR26] Bruchmann K, LaPierre L (2021). Moral foundations predict perceptions of moral permissibility of COVID-19 public health guideline violations in united states university students. Frontiers in Psychology.

[CR27] Cabinet Office. (2021). COVID-19 response—Spring 2021. *Cabinet Office.* Retrieved from https://www.gov.uk/government/publications/covid-19-response-spring-2021/covid-19-response-spring-2021

[CR28] Cave, P., McDonough, J., Williams, M., Miller, P., Ball, S., & Barker, T. (2021). Covid passports and the risks of a two-tier society. *The Guardian*. Retrieved from https://www.theguardian.com/world/2021/apr/07/covid-passports-and-the-risks-of-a-two-tier-society

[CR29] Chan EY (2021). Moral foundations underlying behavioral compliance during the COVID-19 pandemic. Personality and Individual Differences.

[CR30] Chan HF, Brumpton M, Macintyre A, Arapoc J, Savage DA, Skali A, Stadelmann D, Torgler B (2020). How confidence in health care systems affects mobility and compliance during the COVID-19 pandemic. PLoS ONE.

[CR31] Chung WT, Wei K, Lin YR, Wen X, Spiro E, Ahn YY (2016). The dynamics of group risk perception in the US after Paris attacks. Social informatics.

[CR32] Clark C, Davila A, Regis M, Kraus S (2020). Predictors of COVID-19 voluntary compliance behaviors: An international investigation. Global Transitions.

[CR33] Clinton J, Cohen J, Lapinski J, Trussler M (2021). Partisan pandemic: How partisanship and public health concerns affect individuals’ social mobility during COVID-19. Science Advances.

[CR34] Cohen, R. (2021). Persuasion vs. coercion: Vaccine debate in europe heats up. *The New York Times*. Retrieved from https://www.nytimes.com/2021/07/23/world/europe/france-covid-vaccine-coercion.html

[CR35] Conway P, Gawronski B (2013). Deontological and utilitarian inclinations in moral decision making: A process dissociation approach. Journal of Personality and Social Psychology.

[CR36] Crawford, C. (2021). White skin, no masks: Libertarianism, the UK anti-lockdown movement and freedom. *King’s College London*. Retrieved from https://www.kcl.ac.uk/white-skin-no-masks-libertarianism-the-uk-anti-lockdown-movement-and-freedom#_ftnref5

[CR37] Das AK, Abdul Kader Jilani MM, Uddin MS, Uddin MA, Ghosh AK (2021). Fighting ahead: Adoption of social distancing in COVID-19 outbreak through the lens of theory of planned behavior. Journal of Human Behavior in the Social Environment.

[CR38] Davidson, G. (2021). Covid Scotland: Anger mounts over Scotland's vaccine passport scheme amid claims it is 'riddled with holes'. *The Scotsman.* Retrieved from https://www.scotsman.com/news/politics/covid-scotland-anger-mounts-over-scotlands-vaccine-passport-scheme-amid-claims-it-is-riddled-with-holes-3377833

[CR39] Day MV, Fiske ST, Downing EL, Trail TE (2014). Shifting liberal and conservative attitudes using moral foundations theory. Personality and Social Psychology Bulletin.

[CR40] de Figueiredo A, Larson HJ, Reicher SD (2021). The potential impact of vaccine passports on inclination to accept COVID-19 vaccinations in the United Kingdom: Evidence from a large cross-sectional survey and modeling study. EClinicalMedicine.

[CR41] Deane, C., Parker, K., & Gramlich, J. (2021). A year of US public opinion on the coronavirus pandemic. *Pew Research.* Retrieved from https://www.pewresearch.org/2021/03/05/a-year-of-u-s-public-opinion-on-the-coronavirus-pandemic/

[CR42] Delfs, A., & Rogers, I. (2022). Europe heads toward a new normal as final covid curbs unwind. *Bloomberg*. Retrieved from https://www.bloomberg.com/news/articles/2022-02-16/europe-heads-back-to-normal-as-germany-joins-end-of-covid-curbs

[CR43] Department of Health and Social Care, & Hancock, M. (2021). More than 20 million UK adults receive both doses of COVID-19 vaccine. *GOV.UK*. Retrieved from https://www.gov.uk/government/news/more-than-20-million-uk-adults-receive-both-doses-of-covid-19-vaccine

[CR44] Department of Health Northern Ireland. (2021). Northern Ireland’s vaccine certification App set to go live. *Department of Health Northern Ireland*. Retrieved from https://www.health-ni.gov.uk/news/northern-irelands-vaccine-certification-app-set-go-live

[CR45] Díaz R, Cova F (2022). Reactance, morality, and disgust: The relationship between affective dispositions and compliance with official health recommendations during the COVID-19 pandemic. Cognition and Emotion.

[CR46] Dickinson JL, McLeod P, Bloomfield R, Allred S (2016). Which moral foundations predict willingness to make lifestyle changes to avert climate change in the USA?. PLoS ONE.

[CR47] Ekici H, Yücel E, Cesur S (2021). Deciding between moral priorities and COVID-19 avoiding behaviors: A moral foundations vignette study. Current Psychology.

[CR48] Esson, G., & Iredje, R. (2022). Vaccine passports: How does Scotland’s scheme work? *BBC News*. Retrieved from https://www.bbc.co.uk/news/uk-scotland-58422607.

[CR49] Feinberg M, Willer R (2013). The moral roots of environmental attitudes. Psychological Science.

[CR50] Feinberg M, Willer R (2015). From gulf to bridge: When do moral arguments facilitate political influence?. Personality and Social Psychology Bulletin.

[CR51] Feinberg M, Willer R (2019). Moral reframing: A technique for effective and persuasive communication across political divides. Social and Personality Psychology Compass.

[CR52] Fieldhouse, E., Green, J., Evans, G. H. S., van der Eijk, C. J. M., & Prosser, C. (2019). *British election study internet panel waves 1–15*. 10.5255/UKDA-SN-8810-1

[CR53] Fredrickson BL, Tugade MM, Waugh CE, Larkin GR (2003). What good are positive emotions in crisis? A prospective study of resilience and emotions following the terrorist attacks on the United States on September 11th, 2001. Journal of Personality and Social Psychology.

[CR54] Freeman D, Loe BS, Chadwick A, Vaccari C, Waite F, Rosebrock L, Jenner L, Petit A, Lewandowsky S, Vanderslott S, Innocenti S, Larkin M, Giubilini A, Yu L, McShane H, Pollard AJ, Lambe S (2020). COVID-19 vaccine hesitancy in the UK: The Oxford coronavirus explanations, attitudes, and narratives survey (Oceans) II. Psychological Medicine.

[CR55] Gallant AJ, Nicholls LAB, Rasmussen S, Cogan N, Young D, Williams L (2021). Changes in attitudes to vaccination as a result of the COVID-19 pandemic: A longitudinal study of older adults in the UK. PLoS ONE.

[CR56] Gelfand MJ, Jackson JC, Pan X, Nau D, Pieper D, Denison E, Dagher M, Van Lange PA, Chiu CY, Wang M (2021). The relationship between cultural tightness–looseness and COVID-19 cases and deaths: A global analysis. The Lancet Planetary Health.

[CR57] Gibson C (2020). From “social distancing” to “care in connecting”: An emerging organizational research agenda for turbulent times. Academy of Management Discoveries.

[CR58] Glover RJ, Natesan P, Wang J, Rohr D, McAfee-Etheridge L, Booker DD, Bishop J, Lee D, Kildare C, Wu M (2014). Moral rationality and intuition: An exploration of relationships between the defining issues test and the moral foundations questionnaire. Journal of Moral Education.

[CR59] Gostin LO, Hodge JG (2020). US emergency legal responses to novel coronavirus. JAMA.

[CR60] Graham J, Haidt J, Koleva S, Motyl M, Iyer R, Wojick SP, Ditto PH, Devine P, Plant A (2013). Chapter two—Moral foundations theory: The pragmatic validity of moral pluralism. Advances in experimental social psychology.

[CR61] Graham J, Haidt J, Nosek BA (2009). Liberals and conservatives rely on different sets of moral foundations. Journal of Personality and Social Psychology.

[CR62] Graham J, Nosek BA, Haidt J, Iyer R, Koleva S, Ditto PH (2011). Mapping the moral domain. Journal of Personality and Social Psychology.

[CR63] Greenhaus JH, Parasuraman S, Wormley WM (1990). Effects of race on organizational experiences, job performance evaluations, and career outcomes. Academy of Management Journal.

[CR64] Groppo, M. (2021). Covid vaccine passports and privacy rights: Is the trade-off justified? *Carter-Ruck*. Retrieved from https://www.carter-ruck.com/insight/covid-vaccine-passports-and-privacy-rights-is-the-trade-off-justified/

[CR65] Grossin, A., Solomon, E., & Dombey, D. (2021). Europe’s motley Covid passport protesters find unity in liberty. *Financial Times*. Retrieved from https://www.ft.com/content/8e94ff2e-6bf0-442c-be60-385ffc393f53

[CR66] Grover T, Bayraktaroglu E, Mark G, Rho EHR (2019). Moral and affective differences in US immigration policy debate on twitter. Computer Supported Cooperative Work: An International Journal.

[CR67] Gualtieri, T. (2022). Spain calls for debate to consider covid as endemic, like flu. *Bloomberg*. Retrieved from https://www.bloomberg.com/news/articles/2022-01-11/spain-calls-for-debate-to-consider-covid-as-endemic-like-flu

[CR68] Gutierrez R, Giner-Sorolla R (2007). Anger, disgust, and presumption of harm as reactions to taboo-breaking behaviors. Emotion.

[CR69] Haidt J (2001). The emotional dog and its rational tail: A social intuitionist approach to moral judgment. Psychological Review.

[CR70] Hall M, Studdert D (2021). Public views about COVID-19 “immunity passports”. Journal of Law and the Biosciences.

[CR71] Hare, S. (2021). Give pause before you raise a glass to the prospect of a vaccine passport. *The Guardian*. Retrieved from https://www.theguardian.com/commentisfree/2021/mar/28/give-pause-before-you-raise-a-glass-to-prospect-of-vaccine-passports

[CR72] Harper CA, Satchell LP, Fido D, Latzman RD (2021). Functional fear predicts public health compliance in the COVID-19 pandemic. International Journal of Mental Health and Addiction.

[CR73] Hayes A (2014). Multiple mediator models. Introduction to mediation, moderation, and conditional process analysis: A regression-based approach.

[CR74] He L, He C, Reynolds TL, Bai Q, Huang Y, Li C, Zheng K, Chen Y (2021). Why do people oppose mask wearing? A comprehensive analysis of US tweets during the COVID-19 pandemic. Journal of the American Medical Informatics Association.

[CR75] Henrich J, Heine SJ, Norenzayan A (2010). Most people are not WEIRD. Nature.

[CR76] Heung, S., Hung, E., & Cheng, L. (2022). Coronavirus: Hong Kong drops on-arrival PCR tests and vaccine pass, city set to reopen its border with mainland China on January 10 at the earliest. *South China morning post.* Retrieved from https://www.scmp.com/news/hong-kong/health-environment/article/3204782/coronavirus-hong-kong-drop-pcr-tests-arrivals-vaccine-pass-quarantine-requirements-close-contacts

[CR77] Heymann D., Ross, E., & Wallace, J. (2022). The next pandemic—When could it be? *Chatham House.* Retrieved from https://www.chathamhouse.org/2022/02/next-pandemic-when-could-it-be

[CR78] Holland, J., Lee, J., & Iafolla, R. (2021). Big tech unleashes vaccine passports as privacy questions loom. *Bloomberg Law.* Retrieved from https://news.bloomberglaw.com/privacy-and-data-security/big-tech-unleashes-vaccine-passports-as-privacy-questions-loom

[CR79] Holmes, O., & Kierszenbaum, Q. (2021). Green pass: How are Covid vaccine passports working for Israel? *The Guardian*. Retrieved from https://www.theguardian.com/world/2021/feb/28/green-pass-how-are-vaccine-passports-working-in-israel

[CR80] Hoover J, Johnson K, Boghrati R, Graham J, Dehghani M (2018). Moral framing and charitable donation: Integrating exploratory social media analyses and confirmatory experimentation. Collabra: Psychology.

[CR81] Horberg EJ, Oveis C, Keltner D, Cohen AB (2009). Disgust and the moralization of purity. Journal of Personality and Social Psychology.

[CR82] Hornsey MJ, Harris EA, Fielding KS (2018). The psychological roots of anti-vaccination attitudes: A 24-nation investigation. Health Psychology.

[CR83] Ibbetson, C. (2021). Britons still broadly support COVID-19 vaccine passports. *YouGov*. Retrieved from https://yougov.co.uk/topics/politics/articles-reports/2021/08/09/britons-still-broadly-support-covid-19-vaccine-pas

[CR84] IJzerman H, Lewis NA, Przybylski AK, Weinstein N, DeBruine L, Ritchie SJ, Vazire S, Forscher PS, Morey RD, Ivory JD, Anvari F (2020). Use caution when applying behavioural science to policy. Nature Human Behaviour.

[CR85] Iyer R, Koleva S, Graham J, Ditto P, Haidt J (2012). Understanding libertarian morality: The psychological dispositions of self-identified libertarians. PLoS ONE.

[CR86] Jain SS, Jain SP, Li YJ (2022). Sustaining livelihoods or saving lives? Economic system justification in the time of COVID-19. Journal of Business Ethics.

[CR87] Jancenelle VE, Javalgi R (2018). The effect of moral foundations in prosocial crowdfunding. International Small Business Journal: Researching Entrepreneurship.

[CR88] Jost JT, Glaser J, Kruglanski AW, Sulloway FJ (2003). Political conservatism as motivated social cognition. Psychological Bulletin.

[CR89] Jung H, Albarracín D (2021). Concerns for others increase the likelihood of vaccination against influenza and COVID-19 more in sparsely rather than densely populated areas. Proceedings of the National Academy of Sciences.

[CR90] Kalla, T. (2021). Passport or affidavit: How vaccine passports could further divide populations and perpetuate cycles of inequality. *Human Rights Pulse*. Retrieved from https://www.humanrightspulse.com/mastercontentblog/passport-or-affidavit-how-vaccine-passports-could-further-divide-populations-and-perpetuate-cycles-of-inequality

[CR91] Kaplan, J., Vaccaro, A., Henning, M., & Christov-Moore, L. (2021). Moral reframing of messages about mask-wearing during the COVID-19 pandemic. 10.31234/osf.io/gfa5r 10.1038/s41598-023-37075-3 PMC10287646 37349385

[CR92] Kelleher, R. S. (2022). A national vaccine pass has quietly rolled out–and red states are getting on board. *Forbes*. Retrieved from https://www.forbes.com/sites/suzannerowankelleher/2022/02/24/national-vaccine-quietly-rolled-out/

[CR93] Kennedy, H. (2021). The vaccine passport debate reveals fundamental views about how personal data should be used, its role in reproducing inequalities, and the kind of society we want to live in. *London School of Economics and Political Science*. Retrieved from https://blogs.lse.ac.uk/impactofsocialsciences/2021/08/12/the-vaccine-passport-debate-reveals-fundamental-views-about-how-personal-data-should-be-used-its-role-in-reproducing-inequalities-and-the-kind-of-society-we-want-to-live-in/

[CR94] Kidwell B, Farmer A, Hardesty DM (2013). Getting liberals and conservatives to go green: Political ideology and congruent appeals. Journal of Consumer Research.

[CR95] Kirk, I. (2022). How do people around the world feel about vaccine passports? *YouGov*. Retrieved from https://yougov.co.uk/topics/international/articles-reports/2022/02/17/how-do-people-around-world-feel-about-vaccine-pass

[CR96] Kivikangas JM, Fernández-Castilla B, Järvelä S, Ravaja N, Lönnqvist J-E (2021). Moral foundations and political orientation: Systematic review and meta-analysis. Psychological Bulletin.

[CR97] Kohlberg L (1975). The cognitive-developmental approach to moral education. Phi Delta Kappan.

[CR98] Koleva SP, Graham J, Iyer R, Ditto PH, Haidt J (2012). Tracing the threads: How five moral concerns (especially Purity) help explain culture war attitudes. Journal of Research in Personality.

[CR99] Kong DT, Belkin LY (2021). You don’t care for me, so what’s the point for me to care for your business? Negative implications of felt neglect by the employer for employee work meaning and citizenship behaviors amid the COVID-19 pandemic. Journal of Business Ethics.

[CR100] Kuiper ME, de Bruijn AL, Reinders Folmer C, Olthuis E, Brownlee M, Kooistra EB, Fine A, Van Rooij B (2020). The intelligent lockdown: Compliance with COVID-19 mitigation measures in the Netherlands. Amsterdam Law School Research Paper.

[CR101] Kukathas C, Smelser NJ, Baltes PB (2001). Libertarianism. International encyclopedia of the social & behavioral sciences.

[CR102] Kukowski C, Bernecker K, Brandstätter V (2021). Self-control and beliefs surrounding others’ cooperation predict own health-protective behaviors and support for COVID-19 government regulations: Evidence from two European countries. Social Psychological Bulletin.

[CR103] Landmann H, Hess U (2018). Testing moral foundation theory: Are specific moral emotions elicited by specific moral transgressions?. Journal of Moral Education.

[CR104] Ledy, M. N. (2021). Togo becomes one of the first African countries to introduce digital vaccine passports. *Gavi The Vaccine Alliance*. Retrieved from https://www.gavi.org/vaccineswork/togo-becomes-one-first-african-countries-introduce-digital-vaccine-passports

[CR105] Lee, N. T., Lai, S., & Skahill, E. (2021). Vaccine passports underscore the necessity of U.S. privacy legislation. *Brookings Institution*. Retrieved from https://www.brookings.edu/blog/techtank/2021/06/28/vaccine-passports-underscore-the-necessity-of-u-s-privacy-legislation/

[CR106] Lehmann EY, Lehmann LS (2021). Responding to patients who refuse to wear masks during the covid-19 pandemic. Journal of General Internal Medicine.

[CR107] Leonard, B. (2022). Red states putting their stamp on vaccine ‘passports’. *Politico*. Retrieved from https://www.politico.com/newsletters/future-pulse/2022/02/23/red-states-putting-their-stamp-on-vaccine-passports-00010875.

[CR108] Lewandowsky S, Dennis S, Perfors A, Kashima Y, White JP, Garrett P, Little DR, Yesilada M (2021). Public acceptance of privacy-encroaching policies to address the COVID-19 pandemic in the United Kingdom. PLoS ONE.

[CR109] Lindeman, T. (2022). Arrests in Ottawa as Canadian truckers block main bridge to US. *The Guardian*. Retrieved from https://www.theguardian.com/world/2022/feb/08/canadian-truckers-block-bridge-to-us-trudeau

[CR110] Lugonja, B. (2021). The NHS COVID Pass in Wales. *Senedd Research, Welsh Parliament*. Retrieved from https://research.senedd.wales/research-articles/the-nhs-covid-pass-in-wales/

[CR111] Magrath P, Nichter M (2022). Moral framing in health promotion: Lessons from an Indonesian case study. Global Health Promotion.

[CR112] Martuscelli, C., & Roberts, H. (2021). Black flags and crucifixes: Italy vaccine passport protests unite strange bedfellows. *Politico*. Retrieved from https://www.politico.eu/article/italy-vaccine-passport-protest-neo-fascists-green-pass/

[CR113] Master, F., & Siu, T. (2022). Hong Kong “overwhelmed” as COVID infections hit record. *Reuters*. Retrieved from https://www.reuters.com/world/china/hong-kong-leader-says-fifth-covid-wave-has-overwhelmed-citys-capacity-2022-02-14/

[CR115] Menezes, P. N., Simuzingili, M., Debebe, Y. Z., Pivodic, P., & Massiah, E. (2021). What is driving COVID-19 vaccine hesitancy in Sub-Saharan Africa? *World Bank Blogs*. Retrieved from https://blogs.worldbank.org/africacan/what-driving-covid-19-vaccine-hesitancy-sub-saharan-africa

[CR116] Merritt, A. (2021). Anti-vaccine protestors target the streets of Exeter city centre. *Devon Live*. Retrieved from https://www.devonlive.com/news/devon-news/anti-vaccine-protestors-target-streets-6203049

[CR117] Mor Barak ME, Cherin DA, Berkman S (1998). Organizational and personal dimensions in diversity climate. The Journal of Applied Behavioral Science.

[CR118] Muldoon, Z. (2021). 'We're a big industry that is broken': Anger over plans for Covid vaccine passports in nightclubs. *ITV.* Retrieved from https://www.itv.com/news/granada/2021-07-20/anger-over-coronavirus-vaccine-passports-plans-in-nightclubs

[CR119] Murphy J, Vallières F, Bentall RP, Shevlin M, McBride O, Hartman TK, McKay R, Bennett K, Mason L, Gibson-Miller J, Levita L (2021). Psychological characteristics associated with COVID-19 vaccine hesitancy and resistance in Ireland and the United Kingdom. Nature Communications.

[CR120] Nilsson A, Erlandsson A, Västfjäll D (2020). Moral foundations theory and the psychology of charitable giving. European Journal of Personality.

[CR121] O’Boyle EH, Forsyth DR (2021). Individual differences in ethics positions: The EPQ-5. PLoS ONE.

[CR122] Office for National Statistics. (2020). Census 2021 paper questionnaires. *Office for National Statistics*. Retrieved from https://www.ons.gov.uk/census/censustransformationprogramme/questiondevelopment/census2021paperquestionnaires.

[CR123] O’Grady T, Vandegrift D (2019). Moral foundations and decisions to donate bonus to charity: Data from paid online participants in the United States. Data in Brief.

[CR124] Osama T, Razai MS, Majeed A (2021). Covid-19 vaccine passports: Access, equity, and ethics. BMJ.

[CR125] Ozdemir S, Ng S, Chaudhry I, Finkelstein EA (2020). Adoption of preventive behaviour strategies and public perceptions about COVID-19 in Singapore. International Journal of Health Policy and Management.

[CR126] Özdüzen, Ö., Ianosev, B., & Ozgul, B. A. (2021). Freedom or self-interest? Motivations, ideology and visual symbols uniting the anti-lockdown protestors in the UK. *Political Studies Association*. Retrieved from https://www.psa.ac.uk/psa/news/freedom-or-self-interest-motivations-ideology-and-visual-symbols-uniting-anti-lockdown.

[CR127] Palan S, Schitter C (2018). Prolific.ac—A subject pool for online experiments. Journal of Behavioral and Experimental Finance.

[CR128] Park, A., Bryson, C., Clery, E., Curtice, J., & Phillips, M. (2013). *British social attitudes: The 30th report*. Retrieved from www.bsa-30.natcen.ac.uk.

[CR129] Pavlović T, Azevedo F, De K, Riaño-Moreno JC, Maglić M, Gkinopoulos T, Donnelly-Kehoe PA, Payán-Gómez C, Huang G, Kantorowicz J, Birtel MD (2022). Predicting attitudinal and behavioral responses to COVID-19 pandemic using machine learning. PNAS Nexus.

[CR130] Penn M (2021). Statistics say large pandemics are more likely than we thought.

[CR131] Pieterse, L. (2022). South Africa lifts COVID restrictions as infection numbers come down. *COVID Passport*. Retrieved March 7, 2022, from https://www.covidpasscertificate.com/south-africa-covid-passport-in-the-works/

[CR132] Pletzer JL, Balliet D, Joireman J, Kuhlman DM, Voelpel SC, Van Lange PAM (2018). Social value orientation, expectations, and cooperation in social dilemmas: A meta-analysis. European Journal of Personality.

[CR133] Plohl N, Musil B (2020). Modeling compliance with COVID-19 prevention guidelines: The critical role of trust in science. Psychology, Health & Medicine.

[CR134] Porat T, Burnell R, Calvo RA, Ford E, Paudyal P, Baxter WL, Parush A (2021). “Vaccine passports” may backfire: Findings from a cross-sectional study in the UK and Israel on willingness to get vaccinated against COVID-19. Vaccines.

[CR135] Prosser AM, Judge M, Bolderdijk JW, Blackwood L, Kurz T (2020). ‘Distancers’ and ‘non-distancers’? The potential social psychological impact of moralizing COVID-19 mitigating practices on sustained behaviour change. British Journal of Social Psychology.

[CR136] Public Health Scotland. (2022). FitforTravel. *Public Health Scotland.* Retrieved from https://www.fitfortravel.nhs.uk/advice/disease-prevention-advice/yellow-fever

[CR137] Quick BL, Stephenson MT (2007). Further evidence that psychological reactance can be modeled as a combination of anger and negative cognitions. Communication Research.

[CR138] Reimer NK, Atari M, Karimi-Malekabadi F, Trager J, Kennedy B, Graham J, Dehghani M (2022). Moral values predict county-level COVID-19 vaccination rates in the United States. American Psychologist.

[CR139] Reuters. (2022). Hong Kong rolls out vaccine passport and tighter COVID measures. *Reuters*. Retrieved from https://www.reuters.com/world/asia-pacific/hong-kong-rolls-out-vaccine-passport-tighter-covid-measures-2022-02-24/

[CR140] Rimal RN, Lapinski MK (2009). Why health communication is important in public health. Bulletin of the World Health Organization.

[CR141] Rossen I, Hurlstone MJ, Dunlop PD, Lawrence C (2019). Accepters, fence sitters, or rejecters: Moral profiles of vaccination attitudes. Social Science and Medicine.

[CR142] Rozin P, Haidt J, McCauley CR, Lewis M, Haviland-Jones JM, Barrett LF (2008). Disgust. Handbook of emotions.

[CR143] Rozin P, Lowery L, Imada S, Haidt J (1999). The CAD triad hypothesis: A mapping between three moral emotions (contempt, anger, disgust) and three moral codes (community, autonomy, divinity). Journal of Personality and Social Psychology.

[CR144] Russell PS, Giner-Sorolla R (2011). Moral anger is more flexible than moral disgust. Social Psychological and Personality Science.

[CR145] Sahakian, B. J., Langley, C., & Savulescu, J. (2021). Vaccine passports: Why they are good for society. *The Conversation*. Retrieved from https://theconversation.com/vaccine-passports-why-they-are-good-for-society-160419

[CR146] Salerno JM, Peter-Hagene LC (2013). The interactive effect of anger and disgust on moral outrage and judgments. Psychological Science.

[CR147] Satria FB, Khalifa M, Rabrenovic M, Iqbal U (2021). Can digital vaccine passports potentially bring life back to “true-normal”?. Computer Methods and Programs in Biomedicine Update.

[CR148] Schaufeli WB, van Dierendonck D, van Gorp K (1996). Burnout and reciprocity: Towards a dual-level social exchange model. Work & Stress.

[CR149] Schneider AB, Leonard B (2021). From anxiety to control: Mask-wearing, perceived marketplace influence, and emotional well-being during the COVID-19 pandemic. Journal of Consumer Affairs.

[CR150] Sesa, G., Wong, B. L. H., Czabanowska, K., Reid, J., Davidovitch, N., Martin-Moreno, J. M., & Middleton, J. (2021). Covid-19 vaccine passports and vaccine hesitancy: Freedom or control? *The BMJ Opinion*. https://blogs.bmj.com/bmj/2021/03/30/covid-19-vaccine-passports-and-vaccine-hesitancy-freedom-or-control/

[CR151] Sharfstein JM, Callaghan T, Carpiano RM, Sgaier SK, Brewer NT, Galvani AP, Lakshmanan R, McFadden SM, Reiss DR, Salmon DA, Hotez PJ (2021). Uncoupling vaccination from politics: A call to action. The Lancet.

[CR152] Skinner, E. (2017). State vaccination policies: Requirements and exemptions for entering school. *National Conference of State Legislatures*. Retrieved from https://www.ncsl.org/research/health/state-vaccination-policies-requirements-and-exemptions-for-entering-school.aspx 29320811

[CR153] Sleat D, Innes K, Parker I (2021). Are vaccine passports and covid passes a valid alternative to lockdown?. BMJ.

[CR154] Smitham, E., & Glassman, A. (2021). The next pandemic could come soon and be deadlier. *Center for Global Development.* Retrieved from https://www.cgdev.org/blog/the-next-pandemic-could-come-soon-and-be-deadlier

[CR155] Sommerville, E. (2021). Anger on campuses as freshers given wristbands to signify Covid vaccination. *The Telegraph.* Retrieved from https://www.telegraph.co.uk/news/2021/09/28/anger-campuses-freshers-given-wristbands-signify-covid-vaccination/

[CR156] Soroka S, Fournier P, Nir L (2019). Cross-national evidence of a negativity bias in psychophysiological reactions to news. Proceedings of the National Academy of Sciences.

[CR157] Sotis C, Allena M, Reyes R, Romano A (2021). COVID-19 vaccine passport and international traveling: The combined effect of two nudges on Americans’ support for the pass. International Journal of Environmental Research and Public Health.

[CR158] Spring VL, Cameron CD, Cikara M (2018). The upside of outrage. Trends in Cognitive Sciences.

[CR159] Stead M, Ford A, Eadie D, Biggs H, Elliott C, Ussher M, Bedford H, Angus K, Hunt K, MacKintosh AM, Jessop C (2022). A “step too far” or “perfect sense”? A qualitative study of British adults’ views on mandating COVID-19 vaccination and vaccine passports. Vaccine.

[CR160] Steiger RL, Reyna C (2017). Trait contempt, anger, disgust, and moral foundation values. Personality and Individual Differences.

[CR161] Süssenbach P, Rees J, Gollwitzer M (2019). When the going gets tough, individualizers get going: On the relationship between moral foundations and prosociality. Personality and Individual Differences.

[CR162] Syropoulos S, Markowitz EM (2021). Prosocial responses to COVID-19: Examining the role of gratitude, fairness and legacy motives. Personality and Individual Differences.

[CR163] Taber KS (2018). The use of Cronbach’s alpha when developing and reporting research instruments in science education. Research in Science Education.

[CR164] The Economist. (2021). Republicans want to ban businesses from requiring proof of vaccination. *The Economist*. Retrieved from https://www.economist.com/united-states/2021/05/01/republicans-want-to-ban-businesses-from-requiring-proof-of-vaccination

[CR114] The Lancet Respiratory Medicine (2022). Future pandemics: Failing to prepare means preparing to fail. The Lancet Respiratory Medicine.

[CR165] Thornton M, Martin W (2022). Pandemics at work: Convergence of epidemiology and ethics. Business Ethics Quarterly.

[CR166] UK Government and Parliament. (2021). Do not rollout Covid-19 vaccine passports. *Petitions UK Government and Parliament*. Retrieved from https://petition.parliament.uk/petitions/569957

[CR167] USC BCI. (2020). *Video 3 [Video]. YouTube*. Retrieved from https://www.youtube.com/watch?v=RQypElI6yeo

[CR168] van Rooij B, de Bruijn AL, Reinders Folmer C, Kooistra EB, Kuiper ME, Brownlee M, Olthuis E, Fine A (2020). Compliance with COVID-19 mitigation measures in the United States. SSRN Electronic Journal.

[CR169] Voigt K, Nahimana E, Rosenthal A (2021). Flashing red lights: The global implications of COVID-19 vaccination passports. BMJ Global Health.

[CR170] Weaver GR, Reynolds SJ, Brown ME (2014). Moral intuition. Journal of Management.

[CR171] Weber Shandwick. (2020). *The state of corporate reputation in 2020: Everything matters now*. Retrieved February 1, 2022, from https://www.webershandwick.com/wp-content/uploads/2020/01/The-State-of-Corporate-Reputation-in-2020_executive-summary_FINAL.pdf

[CR172] Wilford SH, McBride N, Brooks L, Eke DO, Akintoye S, Owoseni A, Leach T, Flick C, Fisk M, Stacey M (2021). The digital network of networks: Regulatory risk and policy challenges of vaccine passports. European Journal of Risk Regulation.

[CR173] Withers, M. (2021). Are vaccine passports the future of work? *Unleash*. https://www.unleash.ai/future-of-work/are-vaccine-passports-the-future-of-work/

[CR174] Wolsko C (2017). Expanding the range of environmental values: Political orientation, moral foundations, and the common ingroup. Journal of Environmental Psychology.

[CR175] World Health Organization (2022). Tracking SARS-CoV-2 variants.

[CR176] Wright AL, Sonin K, Driscoll J, Wilson J (2020). Poverty and economic dislocation reduce compliance with COVID-19 shelter-in-place protocols. Journal of Economic Behavior & Organization.

[CR177] Yu Y, Lau JTF, Lau MMC (2020). Competing or interactive effect between perceived response efficacy of governmental social distancing behaviors and personal freedom on social distancing behaviors in the Chinese adult general population in Hong Kong. International Journal of Health Policy and Management.

